# Reproducibility and Temporal Structure in Weekly Resting-State fMRI over a Period of 3.5 Years

**DOI:** 10.1371/journal.pone.0140134

**Published:** 2015-10-30

**Authors:** Ann S. Choe, Craig K. Jones, Suresh E. Joel, John Muschelli, Visar Belegu, Brian S. Caffo, Martin A. Lindquist, Peter C. M. van Zijl, James J. Pekar

**Affiliations:** 1 Russell H. Morgan Department of Radiology and Radiological Science, Johns Hopkins University School of Medicine, Baltimore, MD, United States of America; 2 F. M. Kirby Research Center for Functional Brain Imaging, Kennedy Krieger Institute, Baltimore, MD, United States of America; 3 International Center for Spinal Cord Injury, Kennedy Krieger Institute, Baltimore, MD, United States of America; 4 Department of Biostatistics, Bloomberg School of Public Health, Johns Hopkins University, Baltimore, MD, United States of America; 5 Department of Neurology, Johns Hopkins University School of Medicine, Baltimore, MD, United States of America; Max Planck Institute for Human Cognitive and Brain Sciences , GERMANY

## Abstract

Resting-state functional MRI (rs-fMRI) permits study of the brain’s functional networks without requiring participants to perform tasks. Robust changes in such resting state networks (RSNs) have been observed in neurologic disorders, and rs-fMRI outcome measures are candidate biomarkers for monitoring clinical trials, including trials of extended therapeutic interventions for rehabilitation of patients with chronic conditions. In this study, we aim to present a unique longitudinal dataset reporting on a healthy adult subject scanned weekly over 3.5 years and identify rs-fMRI outcome measures appropriate for clinical trials. Accordingly, we assessed the reproducibility, and characterized the temporal structure of, rs-fMRI outcome measures derived using independent component analysis (ICA). Data was compared to a 21-person dataset acquired on the same scanner in order to confirm that the values of the single-subject RSN measures were within the expected range as assessed from the multi-participant dataset. Fourteen RSNs were identified, and the inter-session reproducibility of outcome measures—network spatial map, temporal signal fluctuation magnitude, and between-network connectivity (BNC)–was high, with executive RSNs showing the highest reproducibility. Analysis of the weekly outcome measures also showed that many rs-fMRI outcome measures had a significant linear trend, annual periodicity, and persistence. Such temporal structure was most prominent in spatial map similarity, and least prominent in BNC. High reproducibility supports the candidacy of rs-fMRI outcome measures as biomarkers, but the presence of significant temporal structure needs to be taken into account when such outcome measures are considered as biomarkers for rehabilitation-style therapeutic interventions in chronic conditions.

## Introduction

Functional magnetic resonance imaging (fMRI) can noninvasively reveal the functional organization of the human brain, even in the absence of explicit tasks. Referred to as resting-state functional MRI (rs-fMRI), the method exploits synchronous fluctuations in blood oxygen level dependent (BOLD) signal throughout intrinsic brain functional networks [1]. The ability to study the brain’s functional networks without requiring participants to perform explicit tasks has clinical appeal, as it allows use of an identical protocol for all patients, regardless of cognitive or physical limitations. This is especially important in chronic conditions that affect motor function, and the need for non-invasive and reproducible biomarkers is enhanced by advances in long-term therapeutic interventions for such chronic conditions [2–5]. Further, robust changes in resting state networks (RSNs) have been observed in chronic diseases such as spinal cord injury [6], cerebral palsy [4,5], Parkinson’s disease [7,8], multiple sclerosis [9,10], and stroke [11], indicating that rs-fMRI based network outcome measures have the potential to serve as biomarkers for chronic diseases and their progression, as well as the effects of possible therapeutics.

Here we report the use of a unique longitudinal dataset that covers the time span of 185 weeks with weekly repeat measures. The dataset is exceptional in its length and frequency of acquisition and provides a unique opportunity to gain insight into two different aspects of rs-fMRI derived measures that were previously not accessible: 1) the reproducibility of the RSN outcome measures over an extended time period relevant for long-term clinical trials, and 2) inter-session temporal characteristics of the multi-year time courses of the rs-fMRI based outcome measures, provided through time series analysis.

In this study, we aimed to: 1) present the unique longitudinal dataset reporting on a healthy adult subject scanned weekly over 3.5 years, 2) identify RSN outcome measures appropriate for clinical trials, with high intra-subject inter-session reproducibility over an extended timeframe, and 3) identify potential parameters-of-interest by assessing the existence of temporal structure within RSN outcome measure time courses. To achieve these goals, we first investigated the intra-subject inter-session reproducibility of independent component analysis (ICA)-derived rs-fMRI outcome measures, namely network spatial maps, BOLD temporal signal fluctuation magnitudes, and temporal correlations between pairs of functional networks (between-network connectivity; BNC). We then performed time series analysis on the time courses of the RSN outcome measures to assess their temporal structure.

The RSN outcome measures were stable over the period of 185 weeks, with executive RSNs showing the highest reproducibility. Significant trend, annual periodicity, and persistence existed in the time courses of the outcome measures, suggesting that when such outcome measures are considered as biomarkers for rehabilitation-style therapeutic interventions in chronic conditions, it may be beneficial to take into consideration the temporal structure of the outcome measure.

## Material and Methods

### Participants

The longitudinal single-subject dataset was acquired from a healthy volunteer (40 years of age at time of initial scan; male). A total of 158 sessions of MRI data was acquired on a weekly basis, over a span of 185 weeks. Scans were typically performed on Thursday mornings at 11:30am; in cases of scheduling conflicts, scans were performed on different days of the week and/or times, or skipped, depending on the types of conflicts. The initial image acquisition was performed on the 7^th^ of December, 2009, and the last image acquisition was performed on the 20^th^ of June, 2013. Acquisition dates for each session as well as the dates of the missed scans are reported in the S1 Table.

A publically available multi-participant dataset [12] (referred to as “Kirby-21”; available at http://www.nitrc.org/projects/multimodal), which was acquired using the same rs-fMRI imaging protocol on the same MRI scanner, was used in order to confirm that the values of the single-subject RSN measures are within the expected range as assessed from the multi-participant dataset. The multi-participant dataset is from 21 healthy volunteers (22–61 years, mean 32 years, Male/Female ratio: 11/10).

In order to distinguish the study participant of the longitudinal single-subject study from the participants of the Kirby-21 study, the former will be referred as “the subject” from this point on. The term “participants” will be used inclusively to refer to study participants from both single- and multi-participant studies.

Both the longitudinal single-subject and multi-participant Kirby-21 studies were performed under protocols approved by the Institutional Review Board at Johns Hopkins University School of Medicine. Signed informed consents were obtained from all study participants of the studies.

### Image Acquisition

All participants were scanned on a 3T Philips Achieva scanner (Philips HealthCare, Best, Netherlands).

T1 weighted (T1w) MPRAGE (Magnetization-Prepared Rapid Acquisition Gradient Echo) structural scans were acquired for each session (acquisition time = 6 min, TR/TE/TI = 6.7/3.1/842 ms, resolution = 1x1x1.2 mm^3^, SENSE factor = 2, flip angle = 8°).

Rs-fMRI data of the subject was acquired using a multi-slice SENSE-EPI pulse sequence [13,14] with TR/TE = 2000/30 ms, SENSE factor = 2, flip angle = 75°, 37 axial slices, nominal resolution = 3x3x3 mm^3^, 1 mm gap, 16 channel neuro-vascular coil, number of dynamics (frames) per run = 200. Identical imaging parameters were used to acquire the Kirby-21 rs-fMRI data [12], except for the number of dynamics per run, which was 210. Only the first 200 dynamics of the multi-participant data were analyzed, in order to match the length of the runs with the single-subject data. One of the 21 healthy volunteer dataset was identified to include excess motion and was excluded from further data analysis. The rs-fMRI scans were always acquired after the T1w scans, to allow participants to get acclimated to the noise and environment inside the scanner. Participants were instructed to stay as still as possible with their eyes closed during the entire scan, and no other instruction was provided.

### Data Processing

Preprocessing of the rs-fMRI datasets was performed using SPM8 (http://www.fil.ion.ucl.ac.uk/spm) [15] and Matlab (Natick, MA). The preprocessing pipeline included: 1) slice timing correction, used to correct differences in image acquisition time between image slices, 2) motion correction, 3) co-registration, used to align structural images to functional images, 4) unified segmentation-normalization [16], used to transform functional images to normalized Montreal Neurological Institute (MNI) space (2x2x2 mm^3^), 5) high pass filtering with 0.01 Hz cutoff, used to eliminate slowly varying background noise and effects of scanner drift, and 6) spatial smoothing using 6 mm full-width at half-maximum Gaussian kernel (*i*.*e*., twice the nominal size of the rs-fMRI acquisition voxel), used to suppress noise and reduce effects of imperfect normalization.

Group ICA of fMRI toolbox (GIFT) software (http://mialab.mrn.org/software/gift) [17] was used to perform group independent component analysis (GICA) [18]. Single- and multi-participant datasets were combined, and two steps of principal component analysis (PCA) data reduction were performed for group level analysis, where individual session data were first reduced to 70 principal components. The reduced data was then concatenated in the temporal direction and further reduced to 35 principal components.

Estimation of the number of independent components (*i*.*e*., 35) was guided by order selection using the minimum description length (MDL) criterion [19]. The dimensionality of the individual session PCA data reduction (*i*.*e*., 70) was set by doubling the estimated component number, to ensure robust backreconstruction [20,21] following the ICA decomposition.

ICA [22] is one of the most commonly used methods for analyzing rs-fMRI data. It models the data as a linear mixture of signals originating from spatially-independent sources, and then estimates the sources by maximizing their independence [23]. These sources include not only the spontaneous fluctuations in BOLD signals in functional networks, but also “nuisance” signals such as those arising from head motion, respiration, and cardiac pulsations. Later, these nuisance components are eliminated and only sources identified as RSNs are retained for further analysis. One of the biggest advantages of the method is that it allows the analysis of rs-fMRI data without *a priori* knowledge of the sources [18,24]. In this study, ICA was performed using the InfoMax algorithm [22], and the process yielded a total of 35 aggregate independent component (IC) spatial maps and associated time courses. Single-session maps for each session (single-subject) and participant (multi-participant) were obtained using backreconstruction [18] via the “GICA3” procedure [20]. A flowchart that visualizes the details of the preprocessing and GICA steps is presented as S1 Fig.

RSNs were identified manually from the 35 ICs estimated. Three were rejected due to low reliability of the ICs as assessed using the ICASSO toolbox [25]. The spatial distribution (*i*.*e*., grey matter vs. white matter and cerebral spinal fluid) and temporal frequency power distribution of the remaining 32 ICs were manually assessed, and 18 ICs were eliminated as representing non-neuronal sources such as head motion, respiration, and cardiac pulsations—specifically, the peak activations of the networks were required to be within the gray matter, and RSN spatial maps were required to have low overlap with vascular and ventricular regions. Motion artifact components that display high intensity values around the edges of the brain were also identified and eliminated.–The process identified the remaining 14 ICs as RSNs that represent unique functional networks.

### Resting State Network Outcome Measures

The reproducibility and temporal structure (trend, annual periodicity, and persistence) were assessed for three types of RSN outcome measures: spatial similarity of RSN maps, temporal signal fluctuation magnitude, and BNC.

#### Spatial similarity of RSN maps

The spatial similarity of each week’s RSN spatial maps to the group mean map, as calculated using η^2^ [26], was obtained as an outcome measure. First, the single-session RSN maps for each week were obtained through backreconstruction of the aggregate maps, and converted to z-score using Fisher’s r-to-z transformation [18,20]. A given voxel’s value in each RSN maps, therefore, represents the weight of the RSN time course with respect to the measured relative BOLD signal. The similarity measure η^2^ [26–28] was defined as:

$$\eta^{2}=1-\frac{\sum_{i=1}^{n}{\left(a_{i}-m_{i}\right)}^{2}+{\left(b_{i}-m_{i}\right)}^{2}}{\sum_{i=1}^{n}{\left(a_{i}-\bar{M}\right)}^{2}+{\left(b_{i}-\bar{M}\right)}^{2}}$$ (1)

where *i* represent voxel index within a brain, *n* is number of voxels in a brain, *a*_*i*_ and *b*_*i*_ are the values at position *i* in maps *a* and *b*, respectively, *m*_*i*_ is the mean value of the two images at position *i*, and $\overset{-}{M}$ is the grand mean across the mean image *m*. η^2^ values can range from 0 to 1, where 0 indicates no similarity between two images, and 1 indicates that two images are identical.

By calculating the fraction of the variance in one image accounted for by variance in a second image, η^2^ reports on the difference in the values at corresponding points in the two images. One of the biggest advantages of η^2^ is that it allows the quantification of differences/similarity of the two images instead of the correlational relationship between them [26].

Finally, for each network, a spatial overlap map [29] was created in order to provide visual means to assess the repeatability of the single-session RSN maps. The process first involved thresholding (z-score > 1) of the single session maps to obtain voxels most representative of each RSN. The resulting binary maps were subsequently summed, and then normalized by dividing the maps by the total number of image acquisition, and multiplying by 100 to convert to percentage.

#### Temporal fluctuation magnitude of RSN time courses

The magnitude of temporal signal fluctuations for each RSN was calculated as the quadratic mean (root mean square; RMS) of the backreconstructed time courses that were scaled to the original data to represent percent signal change [18,20].

#### Between-network connectivity of RSN time courses

BNC, a measure of synchrony between RSNs, was computed for each session as the Pearson correlation coefficient of the network time courses [30,31].

### Analysis of RSN Outcome Measures over Sessions

#### Reproducibility

The intra-class correlation (ICC), a metric of test-retest reliability, is widely used in the rs-fMRI reproducibility literature. However, the ICC cannot be used for the present study, which is a longitudinal case report, because there is no ‘class’ (or group) of subjects who underwent 3.5 years of weekly scanning. Instead, for each type of RSN outcome measure (*i*.*e*., spatial similarity of RSN maps, temporal fluctuation magnitude, and BNC), intra-subject inter-session reproducibility was characterized using coefficient of variation (CV), defined as the ratio of standard deviation (SD) to mean, expressed in percentage. CV enables the comparison of data sets with different means, by providing a standardized measure of dispersion. However, the calculated CV can appear artificially inflated if a mean value of a data set is close to zero. Therefore, in order to help keep things in perspective, we also report corresponding SD values.

#### Time series analysis—trend, annual periodicity, and persistence

The single-subject, multi-year acquisition of the longitudinal rs-fMRI dataset enabled time series analysis of weekly RSN outcome measures and observation of their temporal structure (trend, annual periodicity, and persistence) over the extended time period.

Existence of linear trends in the weekly RSN outcome measures was tested using general linear model. Significance of the trend was tested using F statistics, and each outcome measure’s p-value was adjusted for multiple comparisons using false discovery rate (FDR) correction for the number of tested RSNs.

Recognizing that changes in degree of subject motion as well as changes in signal intensity due to variable scanning environment over time may introduce linear trends not of neurological origin, the degree of subject motion over each session over the 185 weeks period was assessed to identify potential confounds. A quantitative measure of subject motion was provided by frame-wise displacement (FD) [32], which was calculated by summing the absolute value of the three differenced translational realignment parameters and the three differenced rotational parameters, which were converted from radians to millimeters by assuming a brain radius of 50 mm.

Additionally, potential changes in signal intensity arising from a variable scanning environment were assessed using a dataset from a concurrent ongoing phantom stability scan within the F.M. Kirby Research Center. The study was run by placing a 15 cm diameter silicone oil filled sphere “phantom” in a 32 channel head coil and running gradient-echo echo-planar imaging (EPI) scans (TR/TE = 3000/40ms, 20 axial slices, imaging matrix: 64x64, field of view: 230 x 230 mm, 3mm slice thickness, 1 mm gap, 300 dynamics) [33], which is equivalent to running a rs-fMRI scan. Signal intensity of the phantom from the corresponding weeks was calculated, and a weekly signal intensity measure was constructed for detection of any linear trend. To prevent reporting of spurious results, permutation tests with 1000 iterations were performed.

The significance of the spectral peaks at 0.0192 weeks^-1^ (annual periodicity; 1/52.18 weeks) in the RSN outcome measures was tested using a procedure for finding spectral peaks in time series described by Ahdesmaki *et al* [34,35] at p < 0.05, after FDR correction for the number of tested RSNs. The robust detection method uses Fisher’s g-test for the detection of periodic fluctuations in multiple time courses. The method also incorporates regression based methods to find the spectral estimate of a time course instead of using the basic periodogram, allowing robust estimation of spectral peaks in non-uniformly sampled data with unknown noise characteristics. This makes the method especially appropriate for the 185 weeks dataset (with 158 time points) described in this study. We refer readers to the above mentioned references for further details of the detection method. The seasonal effect on RSN outcome measures was also investigated by correlating the RSN outcome measure time courses with the recorded daily maximum temperature of Baltimore (freely distributed by the National Oceanic and Atmospheric Administration (NOAA); www.noaa.gov). To prevent reporting of spurious results, permutation tests with 1000 iterations were performed.

Finally, existence of autocorrelation within the RSN outcome measures was assessed by estimating the autoregressive moving average (ARMA) models of the weekly RSN outcome measures using automatic spectral analysis [36,37]. As with the robust spectral peak detection method used to observe annual periodicity, the method is specifically designed to account for non-uniformly sampled data with unknown noise characteristics. ARMA model is estimated in two parts, through separate estimation of the autoregressive (AR) and moving average (MA) models. Traditional ARMA model estimation algorithms often utilize maximum likelihood (ML) approach for both the AR and MA model estimations. However, the ML approach is known to provide poor estimates of the MA model when there are missing data. We therefore utilize the automatic spectral analysis method, which uses reduced statistics algorithm [36,37] to improve estimates of MA model.

## Results

### Functional Networks

**Fig 1:**
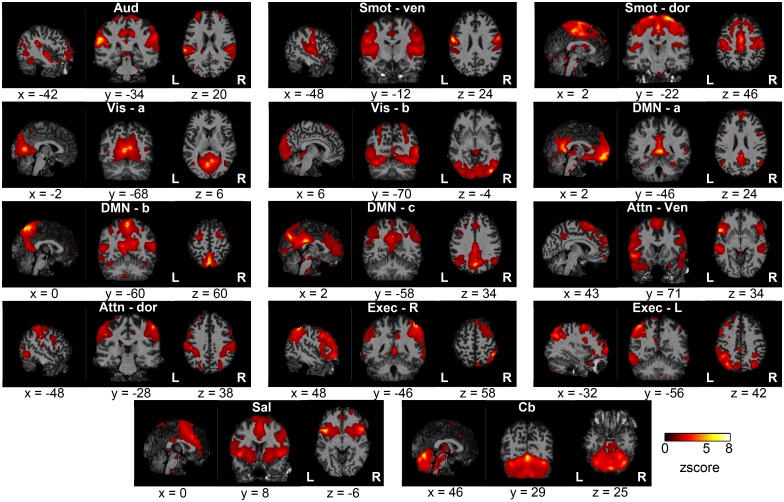
Aggregate spatial maps of the resting state networks (RSNs). Group independent component analysis (GICA) was used to estimate the RSNs and obtain the aggregate spatial maps. The spatial maps of each RSN are shown as subfigures, with representative sagittal, coronal, and axial views (left-to-right) overlaid on structural images within the Montreal Neurological Institute (MNI) template space; coordinates (in mm) for each view are indicated below each subfigure. (Aud: auditory, Smot: seonsorimotor, Vis: visual, DMN: default mode network, Attn: attention, Exec: executive, Sal: salience, Cb: cerebellar, ven: ventral, dor: dorsal, R: right, L: left).

Fourteen RSNs were identified: auditory network (Aud), ventral and dorsal sensorimotor networks (Smot-ven, Smot-dor), two visual networks (Vis-a, Vis-b; arbitrarily labeled), three default mode networks (DMN; DMN-a, DMN-b, DMN-c; arbitrarily labeled), ventral and dorsal attention networks (Attn-ven, Attn-dor), left and right executive-function networks (Exec-L, Exec-R), a salience network (Sal), and a cerebellar network (Cb). Fig 1 shows aggregate spatial maps of the 14 RSNs in representative sagittal, coronal, and axial views.

### Reproducibility

#### Spatial similarity of RSN maps

**Fig 2:**
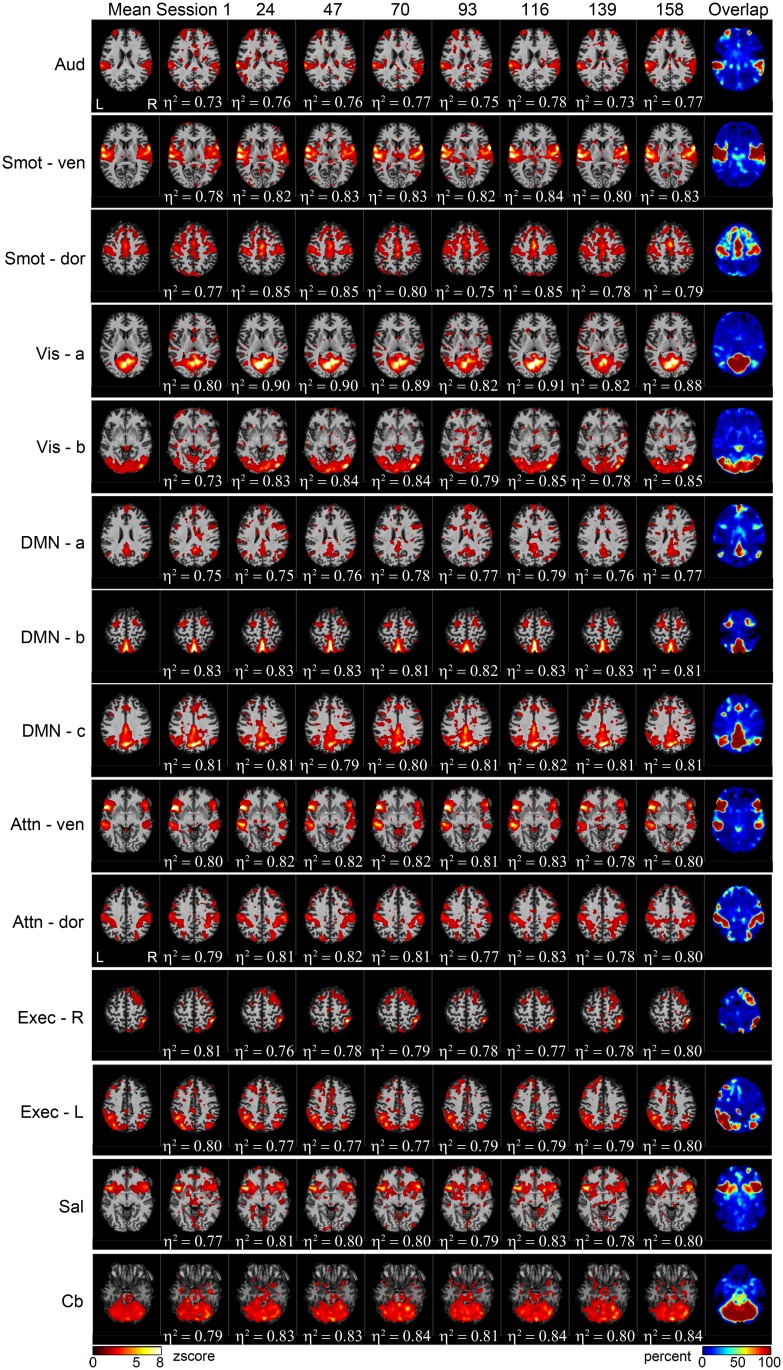
RSN spatial maps for representative weekly sessions. RSN mean spatial maps (leftmost column), representative backreconstructed weekly single-session spatial maps (middle eight columns), and overlap maps (rightmost column) for the 14 RSNs. The degree of spatial similarity of each session’s spatial map to the corresponding mean map, as measured using eta-squared (η^2^), is indicated below the single-session maps.

Backreconstructed, single-session RSN spatial maps from representative imaging sessions and the mean spatial maps of the 14 RSNs are shown in the middle and leftmost column of Fig 2, respectively. Spatial overlap maps, whose values within each voxel represent the fraction of the time the voxel is categorized as a member of the corresponding RSN, are shown in the rightmost columns of Fig 2. Each overlap map showed good agreement with the corresponding group mean spatial map.

**Fig 3:**
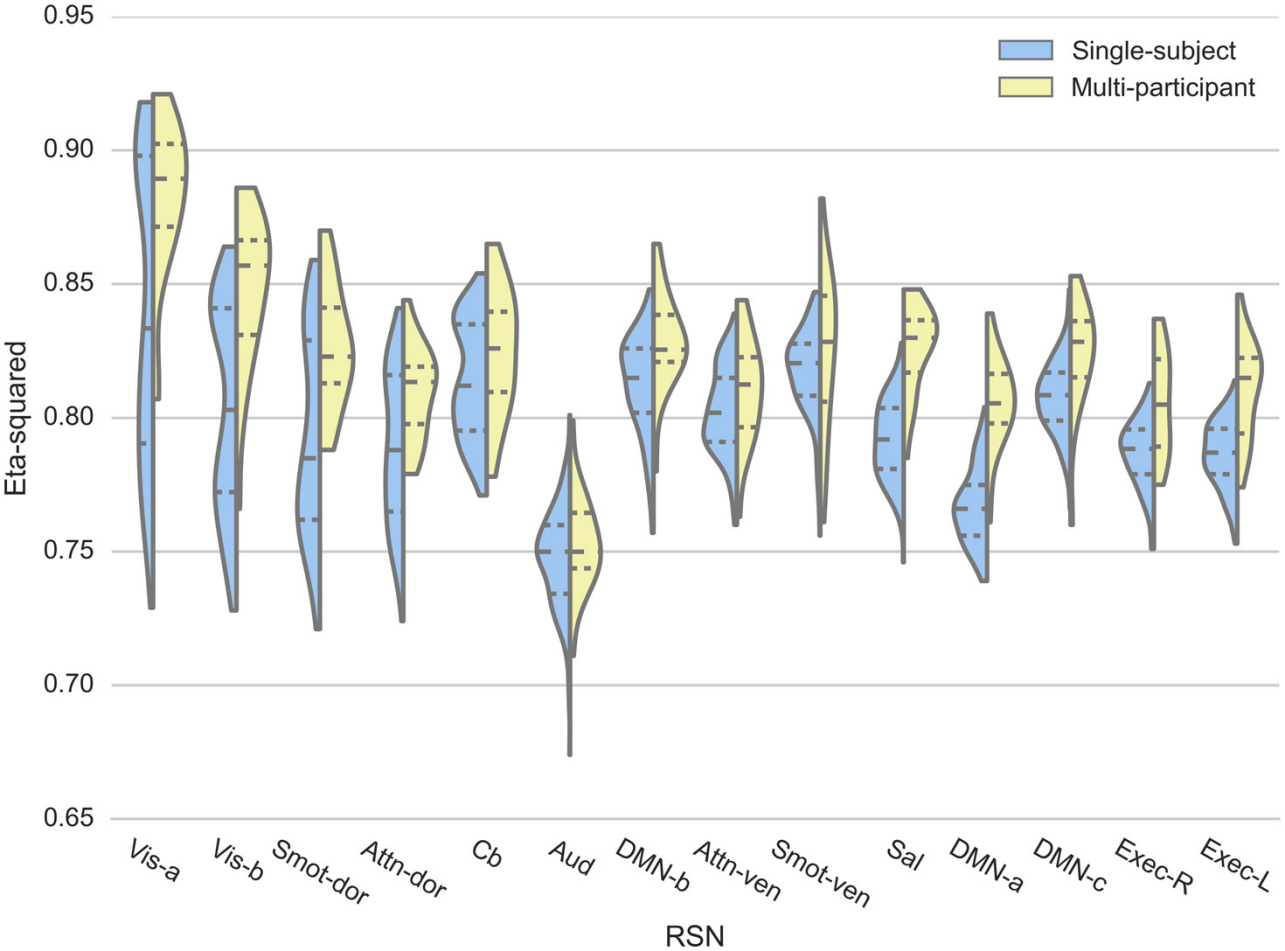
Reproducibility of RSN spatial maps. Spatial similarity of each session’s RSN spatial map to the corresponding group mean map, measured using eta-squared (η^2^), for single-subject (blue) and multi-participant (yellow) datasets, is visualized using violin plots. The first, second, and third quartiles of the data are represented within the violin plots as dotted lines.

Reproducibility of the spatial map similarity measure for both single- and multi-participant datasets is presented using violin plots (Fig 3). For visualization purposes, the violin plots were sorted based on the interquartile range of the single-subject data.

For each RSN, the degree of spatial similarity of the single-subject dataset’s backreconstructed spatial maps to the group mean map was found to be high, with mean η^2^ values ranging from 0.747 to 0.841 (Fig 3, Table 1). Also, for all RSNs, the median (second quartile) η^2^ values of the single-subject dataset were within the range of η^2^ values of the multi-participant dataset.

**Table 1:** Reproducibility of resting state network (RSN) spatial maps.

RSN	Spatial similarity (η^2^) to group mean map					
	Single-subject			Multi-participant		
	mean	SD	CV^¶^	mean	SD	CV
Exec-R	0.786	0.0126	1.60	0.810	0.0183	2.26
Exec-L	0.787	0.0130	1.65	0.806	0.0195	2.42
DMN-c	0.808	0.0138	1.70	0.824	0.0210	2.55
DMN-a	0.767	0.0145	1.89	0.805	0.0177	2.20
Sal	0.792	0.0152	1.91	0.826	0.0166	2.01
Smot-ven	0.817	0.0162	1.98	0.824	0.0306	3.72
Attn-ven	0.802	0.0165	2.05	0.811	0.0203	2.50
DMN-b	0.813	0.0185	2.27	0.828	0.0186	2.25
Aud	0.747	0.0185	2.48	0.753	0.0190	2.52
Cb	0.814	0.0218	2.68	0.824	0.0239	2.90
Attn-dor	0.789	0.0289	3.66	0.809	0.0175	2.16
Smot-dor	0.792	0.0376	4.75	0.826	0.0232	2.81
Vis-b	0.804	0.0391	4.86	0.847	0.0300	3.54
Vis-a	0.841	0.0562	6.68	0.882	0.0304	3.44

^¶^ Sorting column/variable.

The mean, standard deviation (SD), and coefficient of variation (CV) values for each RSN are shown for the single- and multi-participant datasets. (Aud: auditory, Smot: seonsorimotor, Vis: visual, DMN: default mode network, Attn: attention, Exec: executive, Sal: salience, Cb: cerebellar, ven: ventral, dor: dorsal, R: right, L: left)

The two visual networks (Vis-a and Vis-b) and a sensorimotor network (Smot-dor) showed the lowest intra-subject inter-session reproducibility, with CV values of 6.68, 4.86, and 4.75%, while the executive networks (Exec-R and Exec-L) showed the highest reproducibility, with CV values of 1.60 and 1.65% (Table 1). Similarly, the sensorimotor network (Smot-ven) and visual (Vis-b, and Vis-a) networks were also the least reproducible (intra-subject inter-session reproducibility; CV = 3.72, 3.54, 3.44%, respectively) for the multi-participant dataset. Sal network showed the highest inter-subject reproducibility (CV = 1.91%).

A test of equal variance (f-test) indicated that Smot-ven network showed higher intra-subject inter-session spatial map reproducibility compared to its inter-participant reproducibility (p < 0.05, corrected; Table 1). The Vis-a network, on the other hand, showed an opposite trend; intra-subject inter-session reproducibility was significantly lower than the inter-participant reproducibility (p < 0.05, corrected; Table 1). The higher intra-subject inter-session spatial map reproducibility for the Smot-ven network was preserved when the same analysis was performed using only the first twenty sessions of the single-subject data, matching the multi-participant dataset’s number of sessions (not reported separately to avoid overlap). Such was not the case for the Vis-a network; while the trend of lower intra-subject inter-session spatial map reproducibility was still observed, the difference was no longer significant.

#### Temporal fluctuation magnitude of RSN time courses

**Fig 4:**
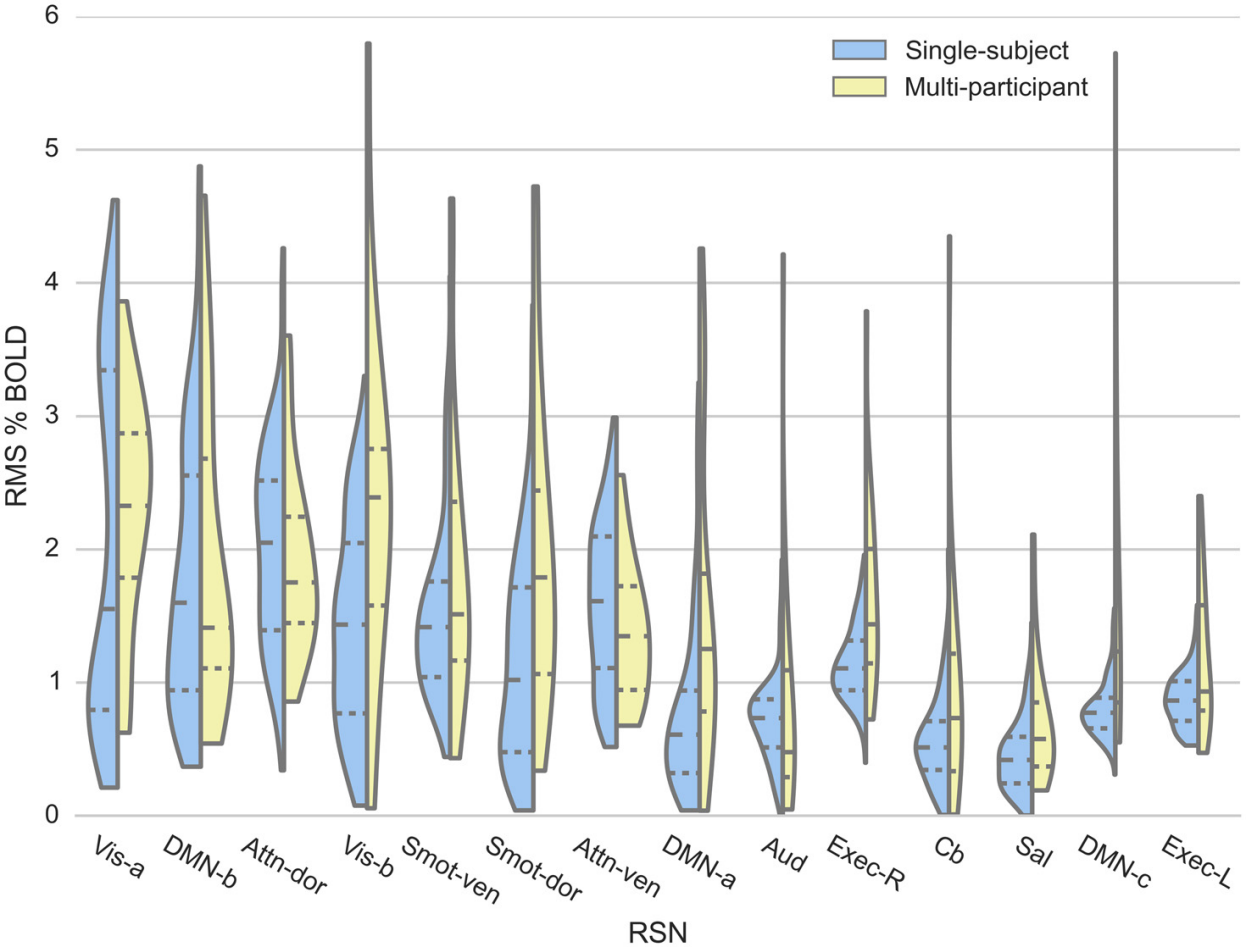
Reproducibility of RSN signal temporal fluctuation magnitude. Blood oxygenation level dependent (BOLD) signal fluctuation magnitude for each session’s RSN time courses, calculated as root-mean-squared (RMS) % BOLD for the single-subject (blue) and multi-participant (yellow) data, is visualized using violin plots.

Reproducibility of the RMS % BOLD value for each session’s backreconstructed time course for both the single- and multi-participant datasets are presented using violin plots in Fig 4. For visualization purposes, the violin plots were sorted based on the interquartile range of the single-subject data.

The single-subject, median temporal signal fluctuation magnitude of each RSN, was within the range of temporal signal fluctuation magnitude values of the same RSNs within the multi-participant dataset, as shown in Fig 4. Also, the ranges of the mean temporal signal fluctuation magnitude for the single- and multiple-participant dataset were similar—from 0.454% to 2.02% and 0.716% to 2.282%, respectively (Table 2).

**Table 2:** Reproducibility of RSN temporal signal fluctuation magnitude.

RSN	Quadratic mean (RMS) of percent signal change					
	Single-subject			Multi-participant		
	mean	SD	CV (%)^¶^	mean	SD	CV (%)
Exec-L	0.879	0.2151	24.5	1.177	0.5682	48.3
Exec-R	1.146	0.2866	25.0	1.646	0.7456	45.3
DMN-c	0.803	0.2153	26.8	1.692	1.3629	80.6
Attn-dor	2.024	0.7637	37.7	1.916	0.7261	37.9
Attn-ven	1.589	0.6157	38.7	1.378	0.5371	39.0
Smot-ven	1.509	0.6486	43.0	1.821	1.1022	60.5
Aud	0.726	0.3308	45.6	0.866	0.9645	111.4
Vis-b	1.428	0.7839	54.9	2.283	1.3495	59.1
DMN-b	1.793	1.0336	57.6	1.902	1.1687	61.4
Cb	0.568	0.3439	60.6	0.948	0.9579	101.0
Sal	0.454	0.2766	60.9	0.716	0.4888	68.2
Vis-a	2.009	1.3182	65.6	2.260	0.9217	40.8
Smot-dor	1.144	0.7834	68.4	1.981	1.2326	62.2
DMN-a	0.783	0.6713	85.8	1.574	1.2306	78.2

^¶^Sorting column/variable.

The mean, SD, and CV values for each network’s temporal signal fluctuation magnitude, expressed as the quadratic mean (root mean square; RMS), is shown for the single- and multi-participant datasets.

For the single-subject dataset, the DMN-a and Smot-dor networks showed the lowest intra-subject inter-session reproducibility over time, with CV values of 85.8 and 68.4%, respectively, while the Exec-L and Exec-R networks showed the highest reproducibility over time, with CV values of 24.5 and 25.0%, respectively (Table 2). For the multi-participant dataset, Aud and Cb networks showed the lowest inter-participant reproducibility with CV values of 111.4 and 101%, respectively, and Attn-dor and Attn-ven networks had the highest reproducibility, with CV values of 37.7 and 38.7%, respectively.

Test of equal variance (f-test) between the single-subject and multi-participant dataset indicated higher intra-subject inter-session reproducibility of temporal fluctuation magnitude, with eight RSN networks (Exec-L, Exec-R, DMN-c, Smot-ven, aud, Vis-b, Cb, and Sal networks) showing significantly higher intra-subject inter-session reproducibility. In contrast, only two RSN networks (Smot-dor and DMN-a) showed significantly lower intra-subject inter-session reproducibility of the networks (Table 2; p < 0.05, corrected). In order to ensure that such high intra-subject inter-session reproducibility is not solely due to the larger sample size of the single-subject dataset, reproducibility was also calculated using the first twenty sessions from the single-subject dataset (not reported separately). The observation was consistent, with six of the eight RSNs (Exec-L, Exec-R, DMN-b, DMN-c, Smot-ven, Aud, and Cb) still showing significantly higher intra-subject inter-session reproducibility (f-test; p < 0.05, corrected)

#### Between-network connectivity of RSNs

Mean BNC values for the single-subject dataset ranged from -0.133 (Sal/DMN-a) to 0.660 (Aud/Smot-ven), and from -0.104 (Sal/DMN-a) to 0.606 (Vis-a/Vis-b) for the multi-participant dataset (Table 3, S2 Table). The top ten RSN pairs with the largest mean BNC values for the single- and multi-participant datasets are reported in Table 3. There was a significant overlap between the top ten lists from the single- and multi-participant datasets, with seven out of ten RSN pairs with the strongest connectivity in the single-subject dataset’s top ten list also appearing in the multi-participant dataset’s top ten list.

**Table 3:** Strength of between-network connectivity (BNC).

RSN pairs	Between network connectivity					
	Single-subject			Multi-participant		
	mean^¶^	SD	CV (%)	mean	SD	CV (%)
Aud / Smot-ven	0.660	0.126	19.1	0.567	0.187	32.9
Smot-dor / Vis-b	0.650	0.135	20.7	0.445	0.223	50.1
Vis-b / Vis-a	0.636	0.166	26.1	0.606	0.191	31.6
Exec-L / Exec-R	0.599	0.0815	13.6	0.509	0.152	29.9
Smot-ven / Vis-a	0.593	0.167	28.1	0.453	0.163	36.0
Vis-b / DMN-b	0.554	0.208	37.6	0.398	0.215	54.2
Smot-dor / Smot-ven	0.551	0.204	37.1	0.554	0.152	27.4
Aud / Vis-a	0.538	0.171	31.8	0.306	0.160	52.2
Smot-dor / Vis-a	0.538	0.212	39.4	0.488	0.182	37.3
Aud / DMN-b	0.527	0.182	34.5	0.345	0.247	71.5
RSN pairs	Between network connectivity					
	Single-subject			Multi-participant		
	mean	SD	CV (%)	mean^¶^	SD	CV (%)
Vis-b / Vis-a	0.636	0.166	26.1	0.606	0.191	31.6
Aud / Smot-ven	0.660	0.126	19.1	0.567	0.187	32.9
Smot-dor / Smot-ven	0.551	0.204	37.1	0.554	0.152	27.4
Exec-L / Exec-R	0.599	0.082	13.6	0.509	0.152	29.9
Smot-dor / Vis-a	0.538	0.212	39.4	0.488	0.182	37.3
Smot-ven / Vis-a	0.593	0.167	28.1	0.453	0.163	36.0
Smot-dor / Vis-b	0.650	0.135	20.7	0.445	0.223	50.1
Vis-b / Attn-dor	0.358	0.233	65.3	0.412	0.230	55.9
DMN-c / DMN-b	0.276	0.198	71.9	0.411	0.208	50.7
Aud / Attn-dor	0.505	0.164	32.5	0.402	0.179	44.5

^¶^Sorting column/variable.

The mean, SD, and CV values of the ten RSN pairs with the largest BNC values of the single- (top table) and multi- (bottom table) participant datasets. Each table was sorted based on the mean BNC values. A full table of mean and SD values for all RSN pairs can be found in the S2 Table.

**Fig 5:**
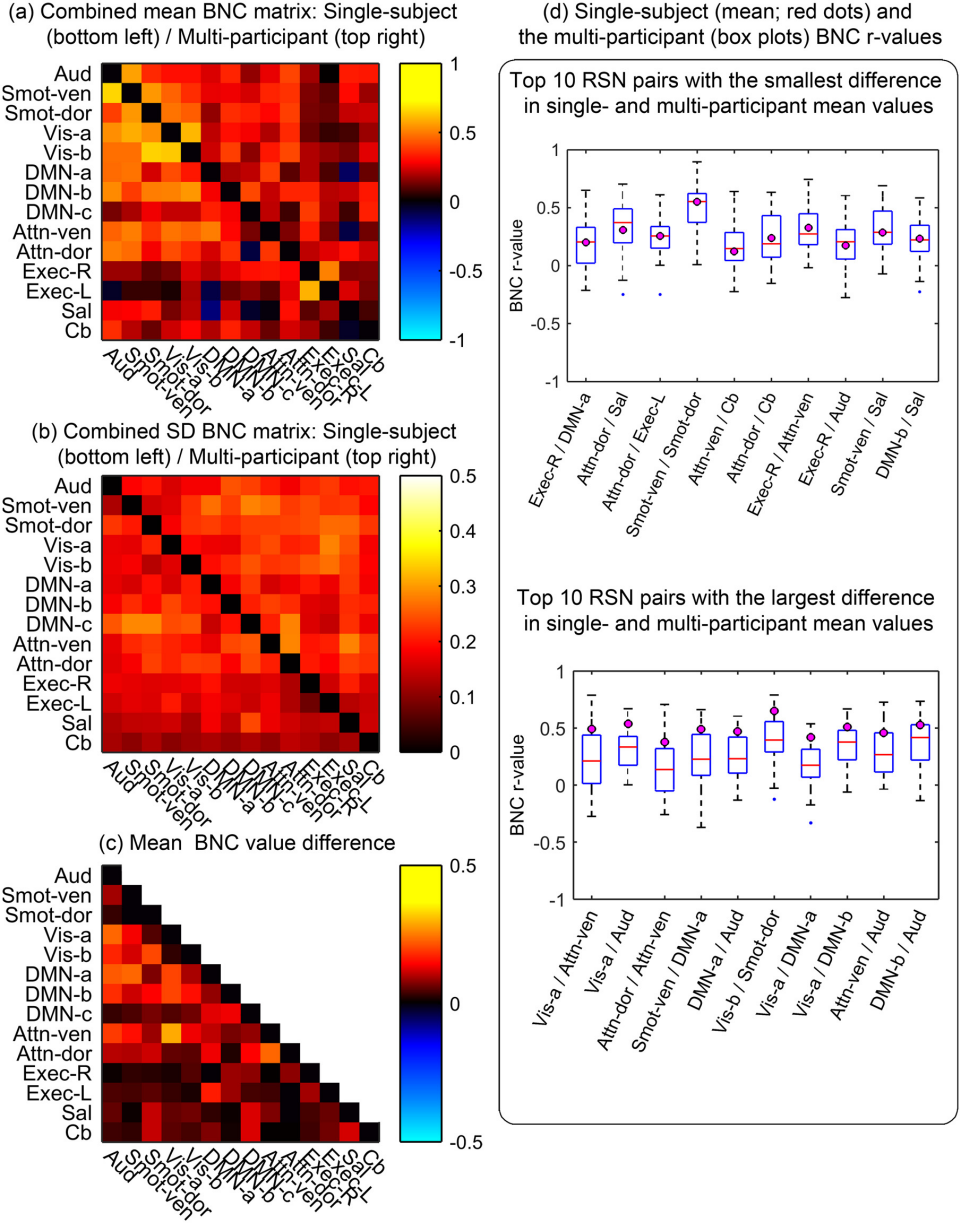
Reproducibility of between-network connectivity (BNC) measurements. The combined BNC matrices show the degree of temporal synchrony between RSN pairs. Mean (a) and standard deviation (SD) (b) BNC values of the single- (below the main diagonal) and multi-participant (above the main diagonal) are shown. The diagonal elements were zeroed for display purposes. (c) Absolute value of the difference between the single- and the multi-participant BNC values. (d) Ten RSN pairs with the smallest (top) and the biggest (bottom) differences between single- and multi-participant mean BNC values. Mean BNC values from the single-subject dataset are overlaid as magenta circles on boxplots reporting on multi-participant data.

Values of mean and SD BNCs are also visualized as matrices in Fig 5(a) and 5(b), respectively. Within each matrix, the single-subject dataset is presented below the main diagonal, and the multi-participant dataset is presented above. For each RSN pair, differences in mean and SD BNC between single- and multi-participant datasets was small, as shown in Fig 5(c).

In order to verify that the mean BNC values for each RSN pair in the single-subject dataset are within the range of BNC values for the corresponding RSN pairs in the multi-participant dataset, the difference in mean BNC between the two datasets were used to sort and identify ten RSN pairs with the smallest (Fig 5d; top) and the largest (Fig 5d; bottom) differences. Fig 5d-bottom shows that the single-subject mean BNC values for the ten RSN pairs with the largest differences are still within the range of the corresponding multi-participant BNC values.

Additionally, CV values of the BNC measures were used to sort and identify ten RSN pairs with the highest reproducibility for both the single- and multi-participant datasets (Table 4). For the single-subject dataset (Table 4; top), the Exec-L/Exec-R network pair was shown to be the most reproducible, with a CV value of 13.6%, while the Smot-dor/Smot-ven network pair was the most reproducible for the multi-participant dataset (Table 4; bottom), with a CV value of 27.4%. For the single-subject dataset, the somatosensory and visual networks had the most reproducible correlations with other RSNs, and this observation also held for the multi-participant dataset.

**Table 4:** Reproducibility of BNC.

RSN pairs	Between network connectivity					
	Single-subject			Multi-participant		
	mean	SD	CV (%)^¶^	mean	SD	CV (%)
Exec-L / Exec-R	0.599	0.0815	13.6	0.509	0.152	29.9
Aud / Smot-ven	0.660	0.126	19.1	0.567	0.187	32.9
Smot-dor / Vis-b	0.650	0.135	20.7	0.445	0.223	50.1
Vis-b / Vis-a	0.636	0.166	26.1	0.606	0.191	31.6
Smot-ven / Vis-a	0.593	0.167	28.1	0.453	0.163	36.0
Aud / Vis-a	0.538	0.171	31.8	0.306	0.160	52.2
DMN-a / Smot-ven	0.489	0.158	32.3	0.259	0.268	104
Cb / DMN-b	0.338	0.110	32.4	0.325	0.209	64.3
Aud / Attn-dor	0.505	0.164	32.5	0.402	0.179	44.5
Aud / Cb	0.360	0.123	34.1	0.323	0.201	62.4
RSN pairs	Between-network connectivity					
	Single-subject			Multi-participant		
	mean	SD	CV (%)	mean	SD	CV (%)^¶^
Smot-dor / Smot-ven	0.551	0.204	37.1	0.554	0.152	27.4
Exec-L / Exec-R	0.599	0.082	13.6	0.509	0.152	29.9
Vis-b / Vis-a	0.636	0.166	26.1	0.606	0.191	31.6
Aud / Smot-ven	0.660	0.126	19.1	0.567	0.187	32.9
Smot-ven / Vis-a	0.593	0.167	28.1	0.453	0.163	36.0
Smot-dor / Vis-a	0.538	0.212	39.4	0.488	0.182	37.3
Attn-ven / DMN-a	0.502	0.172	34.2	0.395	0.154	39.1
DMN-c / Exec-R	0.306	0.151	49.3	0.387	0.162	41.9
Aud / Attn-dor	0.505	0.164	32.5	0.402	0.179	44.5
DMN-b / Exec-R	0.256	0.153	59.8	0.345	0.165	47.9

^¶^Sorting column/variable.

The mean, SD, and CV values of the ten most reproducible network pairs of the single- (top table) and multi-participant (bottom table) datasets. A full table of mean and SD values of all network pairs can be found in the S2 Table.

**Fig 6:**
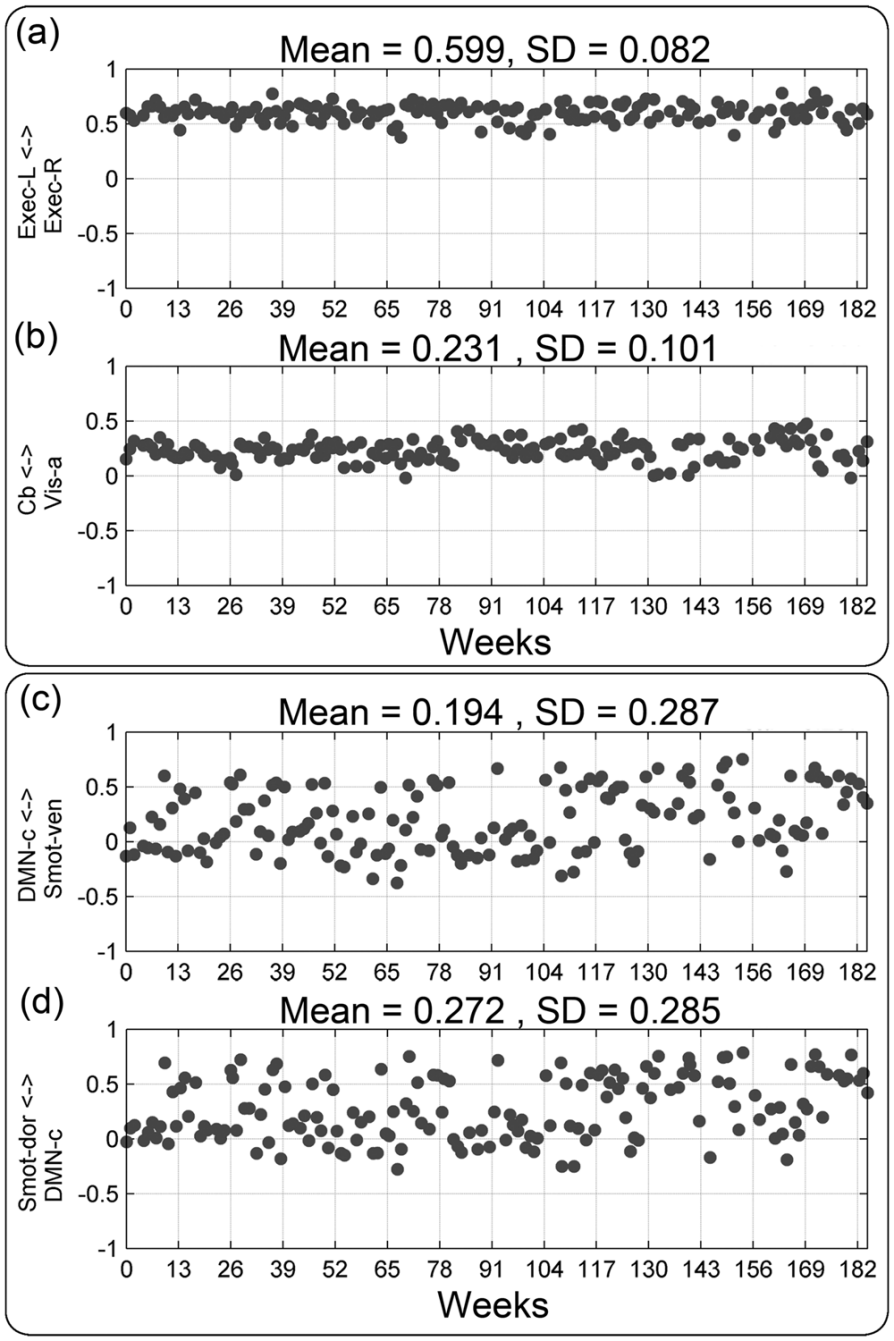
Weekly BNC measures of RSN pairs with the two largest and smallest variations in BNC measurements. Weekly BNC measures are plotted against the corresponding image acquisition weeks for the RSN pairs with the two largest (top) and two smallest (bottom) variations in BNC measurements, as measured by SD.

Finally, recognizing that artificially inflated CV values can arise if a mean value of a data set is close to zero, and therefore to help keep things in perspective, we also report corresponding SD values (Tables 3 and 4, S2 Table, and Fig 5b). BNC SD values ranged from 0.082 to 0.29 (Fig 5b and S2 Table), and the RSN pairs with the smallest SD values in the single-subject dataset were Exec-L/Exec-R and Cb/Vis-a pairs, with SD values of 0.082 and 0.101, respectively. Weekly BNC measures of the two RSN pairs with the smallest SD values (Exec-L/Exec-R and Cb/Vis-a) and two RSN pairs with the largest SD values (DMN-c/Smot-ven and Smot-dor/DMN-c) in the single-subject dataset are showed in Fig 6.

### Time Series Analysis

**Fig 7:**
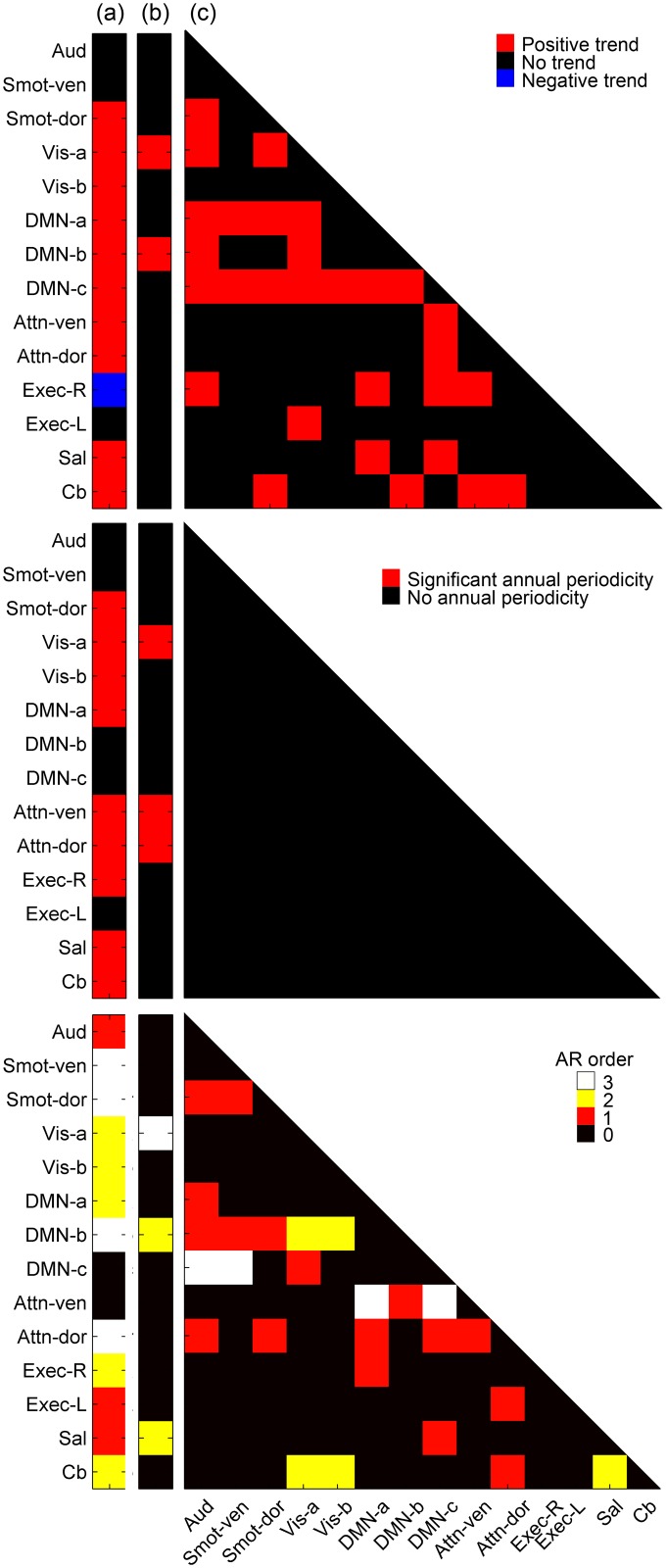
RSNs with significant temporal structures. TOP Existence of significant (after correction for multiple comparisons) linear trends in three RSN outcome measures, namely the (a) spatial similarity (eta-squared, η^2^), (b) temporal signal fluctuation magnitude, and (c) BNC, are visualized using matrices. Red blocks indicate significant positive linear trend, blue blocks negative trend, and black boxes no significant trend. MIDDLE Existence of significant (after correction for multiple comparisons) annual periodicity in three RSN outcome measures. Red blocks indicate significant annual periodicity and black boxes no annual periodicity. BOTTOM AR orders of the estimated ARMA models for RSNs and RSN pairs are visualized for each outcome measures, where black box indicates no autocorrelation, red box AR order of 1, yellow box AR order of 2, and white box AR order of 3. Refer to S3 Table for information on full ARMA model parameters.

Trend. RSNs and RSN pairs with significant (after correction for multiple comparisons) linear trends in rs-fMRI outcome measures are visualized as matrices in the top row of Fig 7. These matrices are color-coded to indicate statistically identified positive, negative, and no trend. For each RSN outcome measure, the intercept and slope of the estimated linear trend, as well as the slope’s corresponding F statistic and associated p-value, are listed in Table 5. Eleven out of the fourteen RSNs showed significant linear trends in η^2^ over 185 weeks. Of these eleven RSNs, ten showed positive trends, while Exec-R showed a negative trend. In comparison, only two (Vis-a and DMN-b) of 14 RSNs showed significant trends in the temporal fluctuation magnitude and twenty-nine out of 105 RSN pairs showed significant trends in BNC. All trends were positive for temporal fluctuation magnitude and BNC. Significant linear trends in BNC were more pronounced in RSN pairs containing DMN-a or DMN-c networks, although significant trends were observed in various RSN pairs involving all categories of functional networks (*i*.*e*., auditory, sensorimotor, visual, DMN, attention, executive, salience, and cerebellar).

**Table 5:** RSNs with significant linear trends in RSN outcome measures.

(a) Eta-squared (η^2^)				
RSN	Intercept	Slope (week^-1^)	F statistic (slope)	p-value
Smot-dor	0.77	1.94E-04	12.78	4.67E-04
Vis-a	0.81	3.77E-04	22.89	3.94E-06
Vis-b	0.78	2.50E-04	20.57	1.14E-05
DMN-a	0.76	1.31E-04	46.92	1.61E-10
DMN-b	0.8	1.15E-04	19.28	2.07E-05
DMN-c	0.8	5.65E-05	7.87	5.67E-03
Attn-ven	0.8	7.40E-05	9.47	2.47E-03
Attn-dor	0.78	1.50E-04	12.91	4.38E-04
Exec-R	0.79	-6.15E-05	11.42	9.16E-04
Sal	0.78	7.76E-05	12.59	5.13E-04
Cb	0.8	1.21E-04	14.96	1.61E-04
(b) Temporal fluctuation magnitude				
RSN	Intercept	Slope (week^-1^)	F statistic (slope)	p-value
Vis-a	1.42	6.51E-03	11.64	8.24E-04
DMN-b	1.4	4.36E-03	8.33	4.46E-03
(c) Between-network connectivity				
RSN pairs	Intercept	Slope (week^-1^)	F statistic (slope)	p-value
Aud/Smot-dor	0.3	1.01E-03	9.4	2.56E-03
Aud/Vis-a	0.46	8.26E-04	11.11	1.07E-03
Aud/DMN-a	0.41	6.44E-04	6.78	1.01E-02
Aud/DMN-b	0.47	6.82E-04	6.48	1.19E-02
Aud/DMN-c	0.02	1.30E-03	13.14	3.90E-04
Aud/Exec-R	0.12	6.50E-04	6.46	1.20E-02
Smot-ven/DMN-a	0.43	6.06E-04	6.79	1.01E-02
Smot-ven/DMN-c	0.06	1.45E-03	12.19	6.24E-04
Smot-dor/Vis-a	0.47	7.68E-04	6.05	1.50E-02
Smot-dor/DMN-a	0.17	7.76E-04	7.27	7.77E-03
Smot-dor/DMN-c	0.13	1.58E-03	14.99	1.59E-04
Smot-dor/Cb	0.07	6.09E-04	10.65	1.36E-03
Vis-a/ DMN-a	0.36	5.88E-04	6.4	1.24E-02
Vis-a/ DMN-b	0.43	8.59E-04	6.01	1.53E-02
Vis-a/ DMN-c	0.13	1.05E-03	8.93	3.27E-03
Vis-a/Exec-L	0.12	-9.81E-04	10.87	1.21E-03
Vis-b/DMN-c	0.09	1.43E-03	16.16	9.02E-05
DMN-a/ DMN-c	0.28	6.38E-04	9.03	3.09E-03
DMN-a/Exec-R	0.13	7.83E-04	7.18	8.16E-03
DMN-a/ Sal	-0.21	8.33E-04	10.34	1.59E-03
DMN-b/ DMN-c	0.19	9.64E-04	11.27	9.90E-04
DMN-b/ Cb	0.29	5.40E-04	11.55	8.60E-04
DMN-c/Attn-ven	-0.02	1.69E-03	23.52	2.96E-06
DMN-c/Attn-dor	-0.17	1.18E-03	11.51	8.78E-04
DMN-c/Exec-R	0.25	6.04E-04	7.44	7.10E-03
DMN-c /Sal	-0.14	1.01E-03	8.3	4.51E-03
Attn-ven/ Cb	0.09	3.82E-04	5.93	1.60E-02
Attn-ven/Exec-R	0.27	5.84E-04	5.89	1.64E-02
Attn-dor/ Cb	0.16	8.40E-04	18.04	3.71E-05

Intercept and slope of the estimated linear trend, as well as the slope’s F statistic and p-value in three RSN outcome measures, namely the (a) spatial similarity (eta-squared, η^2^), (b) temporal fluctuation magnitude, and (c) BNC, for each RSNs with significant linear trends are listed.

The weekly FD (a measure of subject motion) and signal intensity measures from the phantom stability study (a measure of week-to-week scanner stability), did not show significant linear trends (not reported separately). Additionally, permutation tests with 1000 iterations confirmed that significant linear trends no longer exist when weekly outcome measures are randomized.

#### Annual periodicity

RSNs and RSN pairs with significant (after correction for multiple comparisons) annual periodicity in relevant outcome measures are visualized as matrices in the middle row of Fig 7. The matrices are color-coded in black and red, where red blocks highlight RSNs and RSN pairs with significant annual periodicity. Table 6 lists p-values for RSNs and RSN pairs with significant periodicity, for each outcome measure. Nine out of 14 total RSNs showed significant annual periodicity in the η^2^ measure over the period of 185 weeks. In comparison, only three (Vis-a, Attn-ven, and Attn-dor) of the 14 RSNs showed significant annual periodicity for temporal fluctuation magnitude measures, and none of the 105 RSN pairs showed significant annual periodicity for the BNC measures. Additionally, Table 6 shows that the majority of the RSNs with significant annual periodicity also display good correlation with Baltimore’s daily maximum temperature, with correlation coefficients ranging from 0.27 to 0.34. Permutation tests with 1000 iterations confirmed that the observed significant linear trends no longer exist when weekly outcome measures are randomized.

**Table 6:** RSNs with significant annual periodicity and/or significant correlation with daily maximum temperature (Baltimore MD, USA) in outcome measures.

(a) Eta-squared (η^2^)			
RSN	p-value (annual periodicity)	Correlation coefficient (w/ daily maximum temperature)	p-value (w/ daily maximum temperature)
Aud	^&^	0.27	5.44E-04
Smot-dor	1.32E-03	0.31	6.41E-05
Vis-a	2.28E-03	0.29	2.14E-04
Vis-b	4.71E-04	0.29	2.80E-04
DMN-a	3.38E-05	0.29	2.78E-04
Attn-ven	7.44E-03	0.34	1.13E-05
Attn-dor	2.61E-03	0.31	9.74E-05
Exec-R	2.81E-03	^&^	^&^
Sal	3.79E-03	0.31	6.85E-05
Cb	5.29E-04	0.34	1.44E-05
(b) Temporal fluctuation magnitude			
RSN	p-value (annual periodicity)	Correlation coefficient (w/ daily maximum temperature)	p-value (w/ daily maximum temperature)
Vis-a	7.90E-03	0.30	1.43E-04
DMN-b	^&^	0.25	1.44E-03
Attn-ven	7.84E-04	0.30	1.31E-04
Attn-dor	5.72E-03	0.23	3.34E-03

^&^ Statistically not significant.

P-values of RSNs with significant annual periodicity and/or correlation coefficient and associated p-values of RSNs with significant correlation with daily maximum temperature of Baltimore, MD, are listed for two RSN outcome measures; namely the (a) spatial similarity (η^2^) and (b) temporal fluctuation magnitude. No RSN pairs showed significant annual periodicity for BNC measures.

#### Persistence

After ARMA models that best described the autocorrelation structure of RSN outcome measure time courses had been estimated, the order of the AR portion of the model for each RSN and RSN pair were visualized as matrices in the bottom rows of Fig 7 (More detailed description of the ARMA models can be found in S1 Text). The matrices were color-coded, where red indicates AR order of 1, yellow indicates AR order of 2, and white indicates AR order of 3. The MA portion of the model are not visualized, but are listed in S3 Table, which lists the ARMA models that best describe the autocorrelation structure of the weekly outcome measures.

Twelve out of 14 RSNs displayed significant autocorrelation in the η^2^ measure over the period of 185 weeks. In comparison, only three (Vis-a, DMN-b, and Sal) out of 14 RSNs displayed significant autocorrelation for temporal fluctuation magnitude measures, and 26 of the 105 RSN pairs displayed significant autocorrelation in BNC. The order of estimated ARMA models was shown to vary, where AR and MA orders ranged from 0 to 3 for the three types of RSN outcome measures (S3 Table).

## Discussion

Previous reproducibility studies of intrinsic functional networks showed that RSNs are reproducible across participants [29,38,39], within participants over durations of weeks to months [29,40,41], as well as up to a year [42]. The primary goal of this study was to investigate whether intra-subject inter-session RSN outcome measures were reproducible over an even longer period that is more relevant for rehabilitation studies, and this is confirmed by the results.

### Functional Networks

The 14 RSNs identified in this study (Fig 1) were in good correspondence with those consistently reported in previous rs-fMRI studies. The identified RSNs included an auditory network [41], ventral and dorsal sensorimotor networks [1,24,41], two visual networks [24,41], three default mode networks [24,41,43], ventral and dorsal attention networks [41], left and right executive-function networks [44], a salience network [45], and a cerebellar network [29,46]. Of the 35 initially estimated ICs, 21 were rejected as nuisance components (of non-neuronal sources); this rate of rejection of nuisance components was consistent with previous studies [29].

### Reproducibility

#### Spatial similarity of RSN maps

As group mean maps (Fig 2; left column) are obtained by averaging multiple RSN spatial maps across sessions, the most robust “core” activation regions in the RSN spatial maps are preserved within the group mean maps. Measuring the spatial similarity of each backreconstructed RSN map to its corresponding group mean map, therefore, may provide insight into the degree of robustness of core activation region, reflected by the value of η^2^, and the degree of robustness of “non-core” activations, reflected by the variance of η^2^. The high mean η^2^ values of all RSNs, ranging from 0.747 to 0.84, show that the 14 RSNs identified in the study have very robust core activation regions. Stronger core activation regions, however, did not translate to higher reproducibility. The Vis-a network, for example, showed that a network can have a strong core activation region and also have variable non-core activation regions that lead to increased variability in the spatial similarity measure (Table 1 and Fig 3). This network’s variable non-core activation regions can also be visually observed in Fig 2.

For both single- and multi-participant datasets, the three networks with the lowest spatial map inter-session reproducibility were exteroceptive in nature (*i*.*e*., related to the external world), namely visual and sensorimotor networks (Vis-a, Vis-b, Smot-dor, and Cb). Consistent with Kosslyn’s reports on ideation [47], “mind wandering” during rest may have led to increased modulation in the exteroceptive networks.

Finally, for nine out of 14 RSNs, the intra-subject inter-session reproducibility, as measured using CV, of the RSN spatial maps was higher than, or similar to, inter-participant reproducibility, with Smot-ven network displaying significant difference after Bonferroni correction for multiple comparisons. While this observation may be explained by the inherent higher inter-participant variability in the dataset, such a difference could also arise due to imperfect spatial normalization across participants. This suggests that caution may be warranted in using atlas-based seed placement for seed-based correlation, and that more sophisticated spatial normalization methods (*e*.*g*., large deformation diffeomorphic metric mapping (LDDMM) [48,49]) may be beneficial.

#### Temporal fluctuation magnitudes of RSN time courses

For all RSNs, mean intra-subject inter-session RMS % BOLD values (Fig 4, Table 2) were comparable to those reported in a previous multi-participant rs-fMRI study [41]. Additionally, RSNs that showed low spatial map intra-subject inter-session reproducibility also tended to show low temporal fluctuation magnitude intra-subject inter-session reproducibility. Many such least-reproducible RSNs were of exteroceptive nature, and thus may support the previously stated hypothesis that “mind wandering” [47] during rest may have led to increased modulation in exteroceptive networks.

It should also be noted that compared to the CV values of the network spatial maps (Table 1), the CV values of the temporal fluctuation magnitude (Table 2) were significantly larger, ranging from 24.5 to 85.8% for the single-subject dataset and from 37.9 to 111.4% for the multi-participant dataset. These significantly larger CV values were driven by the small mean values and large SD values of the temporal signal fluctuation measurement. Using CV as a measure of dispersion is suboptimal when a mean value is close to zero, as calculated CV measure becomes sensitive to small changes. In this case, a larger issue may be the relatively large SD values of the RSN temporal fluctuation magnitude; the fact that the SD values of many RSNs are close to the corresponding mean values indicates that the measure may be relatively insensitive to small effect sizes, and caution is thus warranted when using the this outcome measure in longitudinal studies.

#### Between-network connectivity of RSN time courses

The ranges of mean BNC values for the single-subject dataset (-0.133–0.660), and the multi-participant dataset (-0.104–0.606) (Table 3, S2 Table) were similar, and strong BNC values were observed between networks within the same functional domains; such as between the Vis-a/Vis-b (0.636), Exec-L/Exec-R (0.599), and Smot-dor/Smot-ven (0.551) RSN pairs. The same held for the multi-participant dataset, with BNC values of 0.606 for Vis-a/Vis-b, 0.554 for Smot-dor/Smot-ven, and 0.509 for Exec-L/Exec-R network pairs. Overall, the mean BNC values for the single- and multi-participant datasets were similar, reflected by the highly symmetric combined correlation matrix of the datasets (Fig 5a). In addition, network pairs that showed strong connectivity within the single-subject dataset also showed strong connectivity within the multi-participant dataset.

Similar to the large CV values that were observed for temporal fluctuation magnitude measurements, the CV values for the BNC measurements were also relatively high, driven by the small mean BNC values. Nonetheless, the intra-subjection inter-session reproducibility of BNC values was good, reflected by the small SD values, as shown in Fig 5b and S2 Table. It should be noted, however, that in cases where a mean BNC value is close to zero, the corresponding SD value needs to be very small before the BNC value of a particular functional network pair can be used as a reproducible longitudinal measure. Therefore, caution should be taken when investigating network pairs with high variance, relative to the corresponding mean BNC value.

For both the single- and multi-participant datasets, RSN pairs with connections to the somatosensory and visual networks demonstrated higher reproducibility (Table 4). This observation agrees with results from a previous study that reported robust inter-participant reliability for the primary visual network [50], compared with other networks.

One of the main observations of this study was the very high intra-subject inter-session reproducibility of the Exec-L and Exec-R networks. For the single-subject dataset, these networks were the most reproducible RSNs for spatial map (Table 1) and temporal signal fluctuation magnitude (Table 2) outcome measures. Additionally, connectivity between the Exec-L/Exec-R RSN pair was the most reproducible, with the smallest CV value of 13.6% (Fig 4, Table 4). This RSN pair was also among the most reproducible networks for all rs-fMRI outcome measures in the multi-participant dataset. Such high intra-subject inter-session reproducibility of the executive control network components has an important implication, as the executive control network is known to be actively involved in functions such as impulse control [51] and consciousness [52,53], and is closely related to disease states such as substance abuse [51], unresponsive wakefulness syndrome [52], and Alzheimer’s disease [54]. Accordingly, this suggests that the executive RSN components and the related outcome measures have high potential for serving as biomarkers of disease states.

### Time Series Analysis

#### Trend

η^2^ values of weekly RSN spatial maps correspond to degrees of spatial similarity between weekly maps and a group mean map. Therefore, the existence of a trend in the η^2^ measurements (Fig 7 TOP(a), Table 5a) is noteworthy. Specifically, a positive trend in η^2^ indicates that as time passes, weekly single-session maps become more stable, looking more like the group mean map. A negative trend, on the other hand, would indicate that weekly maps become more variable, looking less like the group mean map as time passes. Previous studies show that a constant and linear decrease of gray matter volume can be initiated as early as the 20’s [55–57]. While the exact cause of this decline is still under debate, one theory suggests that the decline is due to neuronal and synaptic pruning in the human cortex during reorganization following neural maturation [58]. Our observation of positive trends in the η^2^ values for most of the RSNs may partially be explained by such neuronal pruning of gray matter. Preliminary analysis of the subject’s high resolution T1w structural images revealed that the subject’s gray matter volume decreased by about 0.30 ml/month over the study period [59]; this linear decrease in gray matter volume is consistent with previous reports [55].

Possible long-term habituation of the subject to the scanning environment, and a subsequent decrease of subject motion, was also identified as possible causes for the almost ubiquitous positive trends in η^2^ measures. Analysis of the degree of subject motion, represented by weekly FD measures, however, showed that there was no significant trend in the degree of movement across imaging sessions. Similarly, there was no significant trend in the weekly signal intensity measures from the phantom stability data, indicating that any contribution of variable scanning environment over the 185 weeks to the observed linear trends in the η^2^ values was minimal.

The effect of age on RSNs has been extensively explored in previous literature [20,60–68]. Specifically, the level of coherent activity and degree of co-activation within RSNs are shown to increase during early childhood and young adulthood, indicating neuronal maturation of the networks. This phase is followed by decreases in the level of coherent activity and degree of co-activation in the older population, marked by subsequent cognitive decline [20,60–68]. Such decreases in coherent activity, measured using the magnitude of temporal fluctuation, and degree of co-activation, measured using BNC, were not observed in the subject. Instead, all significant trends reported in this study were positive (Fig 7 TOP(b-c) and Table 5b and 5c). One reason for the discrepancy may be that majority of the above-mentioned studies compared cohorts of young participants (10–34 yrs) and much older participants (60–93 yrs), thus not including the age range of the subject studied here (ca. 40 yrs). Two studies that did include participants in their 40’s [20,68] reported mixed results for different RSNs. It should also be noted that the effects of age on functional connectivity could be mediated by various factors such as stress, education, and exercise [69,70].

A limitation of this study is that the existence of a trend was inferred only using general linear model. This method reports the best fitting linear model of the type specified, but more sophisticated trend detection algorithms may reveal more complex types of trends. Also, it should be noted that effect sizes in the temporal analyses are very small, which may indicate that observed significance is driven by the large number of degrees of freedom. And while this data set is large for fMRI, it is not an exceptionally long time series relative to the norm of seasonal studies.

#### Annual periodicity

The unique longitudinal dataset of the study enabled us to investigate whether seasonal (more specifically, annual) patterns exists in RSN outcome measures, and the results show that such seasonal patterns exist in the weekly η^2^ and temporal fluctuation magnitude measures of relevant RSNs (Fig 7 MIDDLE and Table 6). This result is also confirmed by the good correlation between the RSN outcome measure time courses and Baltimore’s daily maximum temperature observed in Table 6. However, the cause and mechanism of the observed seasonal patterns is unknown at this time. There are, however, several studies that look into the fluctuating patterns of shorter timeframe (*e*.*g*., diurnal and monthly) in functional connectivity measures, and we looked to see whether such higher frequency fluctuation in relevant RSNs translate to lower frequency annual fluctuation.

Interaction between circadian rhythmicity and time awake (homeostatic process), and the resulting diurnal rhythms within various biological systems that range from gene expression [71,72] to body temperature [73] is well-known. Diurnal rhythms were also shown to affect higher order cognitive functions [74–76], and recent studies have shown that diurnal rhythms also exist in the strength of functional connectivity [77–79]. One study in particular showed that highly rhythmic connectivity patterns exist within sub-systems of DMN and sensorimotor network [77]. However, the observed diurnal rhythm in DMN and sensorimotor networks did not translate to the existence of annual periodicity in the same RSNs (*i*.*e*., DMN and sensorimotor networks did not show annual periodicity; Fig 7 TOP(b)). None of the BNC measures display significant annual periodicity (Fig 7 MIDDLE(c) and Table 6c), and this result is consistent in part with a previous study [80], which reported a lack of monthly fluctuation of BNC values. It is not clear at this time why some RSN outcome measures, but not others, show annual periodicity.

#### Persistence

The persistence, or autocorrelation, of a system describes the system’s tendency to stay in the same state from one observation to the next, and is a common feature of many biological systems. While the existence of persistence within a system can complicate the understanding of its underlying mechanism by reducing the number of independent variables and introducing multiple confounding parameters that are not easily separable, persistence can also be exploited to predict future observations based on those of the past [81,82]. Realization of the existence of persistence in a system, and the subsequent estimation of a best-fit mathematical model of the persistence, therefore can lead to: 1) better understanding of the system by quantitatively assessing the fraction of the system’s variance explained by the measured persistence, and 2) better prediction of the behavior of a time course based on past observations. Our results show that persistence is a characteristic of many RSNs and RSN pairs, for all three types of RSN outcome measures (Fig 7 BOTTOM and S3 Table). Within the context of using rs-fMRI derived outcome measures as patient-specific biomarkers of recovery during clinical trials, this observation may lead to the development of more accurate inferences from such data.

### Limitations

A major limitation of this report is that only one subject underwent the weekly scanning for 185 weeks. Scanning additional participants would have helped to ensure the generality of our findings. However, ensuring compliance of multiple participants over such a prolonged period would have been difficult. We have sought to address this issue by including results from a previously acquired multi-participant dataset on the same scanner and with identical acquisition parameters [12], and ensured that the healthy male individual of the longitudinal dataset was a representative healthy control. Estimated means of all network outcome measures for the longitudinal dataset—spatial maps, temporal fluctuation magnitude, and BNC—were within the range of the corresponding outcome measures from the multi-participant dataset, as shown in Figs 3–5 –*i*.*e*., in all cases, values for the longitudinal dataset were within the range of those computed for the multi-participant dataset.

While we report some outcome measures for the multi-participant dataset, we refrain from an in-depth analysis of it, as the focus of this paper was on long-term reproducibility of the single-subject data. However, published reproducibility studies have consistently shown that rs-fMRI derived outcome measures were robust across participants [29,38,39,83], and here we briefly summarize previous literature on this subject. An early study by Chen *et al*., acquired data from 14 healthy participants over the period of 16 days and reported that the intrinsic networks were consistent across multiple sessions [29]. Meindl *et al*., assessed the reproducibility of DMN networks across multiple sessions in 18 healthy participants each scanned three times over the period of a week, and found DMN networks to be highly spatially consistent across sessions [38]. Shehzad *et al*. reported modest to high inter-participant reproducibility in a dataset acquired from 26 participants at three different times over five months [83].

Another limitation of this study is that we report only on outcome measures derived using group ICA (GICA) [18]. However, as one of the most commonly used methods to estimate RSNs, GICA offers many advantages. GICA is an extension of the ICA method, in which reducing and concatenating multi-session/multi-participant fMRI data allows ICA to be applied once to the aggregate data. This obviates the cumbersome and potentially inaccurate matching of components estimated separately from data from individual sessions and/or participants. Furthermore, the alternative method of performing ICA separately for each session may result in different numbers of components for different sessions, depending on the level of noise. A possible consequence of such approach is that networks may split into varying numbers of sub-networks, as seen in higher-order ICA analysis [20]. This in turn introduces additional uncertainty into the network identification process, and may make group inference across sessions unfeasible. Alternatively, use of GICA [18] allows delineation of identical networks for the single- and multi-participant datasets, eliminating the need to perform ICA separately on each dataset. There have been concerns that the identification of aggregate networks using a single dataset of multiple groups, and then backreconstructing the single-session datasets, may bias the results towards the group mean. However, the original report by Calhoun *et al*., [18], and a study by Schmidhorst and Holland [84] show that GICA can identify a network present in as little as 10% of the study population. Also, the findings reported in this study are consistent with reports of the reproducibility of rs-fMRI outcome measures estimated using alternative analytic approaches such as seed based temporal correlation [85–89].

Finally, due to time constraints, additional physiological or psychological measures were not obtained during the acquisition of this dataset. Together, such additional measures would have provided valuable information regarding the nature of the temporal structure we have observed in this study. Indeed, there are now ongoing endeavors to acquire longitudinal rs-fMRI data like ours, augmented with the regular parallel acquisition of many auxiliary measures, ranging from sleep data to blood samples. One such example is the MyConnectome project (http://myconnectome.org/wp/), during which along with rs-fMRI, biological samples (*i*.*e*., blood) as well as data about daily life activities were collected. We expect our study, along with other unique longitudinal studies, will provide new insight into understanding the dynamics of brain function over time.

## Conclusions

Rs-fMRI allows noninvasive observation of brain networks, and has potential to yield biomarkers for clinical trials in neurological diseases where such RSNs may change. The goal of this study was to present a unique longitudinal dataset reporting on a healthy adult subject scanned weekly over 3.5 years, and identify RSN outcome measures with high intra-subject inter-session reproducibility over prolonged timeframes appropriate for rehabilitation trials. ICA was used to identify fourteen RSNs that represent unique functional networks. Three types of rs-fMRI outcome measures, namely spatial map similarity, temporal fluctuation magnitude, and BNC, were found to be reproducible across the extended study period. In particular, the Exec-R and Exec-L networks, which are closely related to disease states such as substance abuse and Alzheimer’s disease, showed high intra-subject inter-session reproducibility for all three types of RSN outcome measures, suggesting that these networks may be of particular interest.

Additionally, we sought to identify potential parameters-of-interest for clinical studies, by assessing the existence of temporal structure in the three types of rs-fMRI outcomes measures. Time series analysis showed that the RSN outcome measures displayed properties including linear trend, annual periodicity, and persistence. This finding suggests that when RSN outcome measures are considered as imaging biomarkers for lengthy therapeutic interventions in chronic conditions it may be beneficial to take the temporal structure parameters into consideration.

## Supporting Information

S1 Fig Preprocessing and group independent component analysis (GICA) flowchart. (DOCX)

S2 Fig Reproducibility of resting state network (RSN) spatial maps, visualized using boxplots. Spatial similarity of each session’s RSN spatial map to the corresponding group mean map, measured using eta-squared (η^2^), for single-subject (a) and multi-participant (b) datasets, is visualized using box plots (end of boxes: quartiles, bar within boxes: median, small dots: outliers). In (b), for each RSN, the mean η^2^ of the single-subject dataset is overlaid as a large gray circle.
(DOCX)

S3 Fig Reproducibility of RSN signal temporal fluctuation magnitude, visualized using boxplots. Blood oxygenation level dependent (BOLD) signal fluctuation magnitude for each session’s RSN time courses, calculated as root-mean-squared (RMS) % BOLD for single-subject (a) and multi-participant (b) datasets, is visualized using boxplots. In (b), for each RSN, the mean RMS % BOLD value for the single-subject dataset is overlaid as a large gray circle.
(DOCX)

S1 Table Acquisition dates of the 158 resting state functional MRI (rs-fMRI) scans. A total of 158 scans were acquired over the period of 185 weeks.
(DOCX)

S2 Table Reproducibility of single- and multi-participant between-network connectivity (BNC) measurements. The mean and standard deviation (SD) values for all resting state network (RSN) pairs are shown for the single- and multi-participant datasets. (Aud: auditory, Smot: seonsorimotor, Vis: visual, DMN: default mode network, Attn: attention, Exec: executive, Sal: salience, Cb: cerebellar, ven: ventral, dor: dorsal, R: right, L: left).
(DOCX)

S3 TableEstimated autoregressive moving average (ARMA) models of each RSNs, for three rs-fMRI outcome measures–η^2^, temporal signal fluctuation, and BNC.Properties of the estimated ARMA models for three outcome measures of each RSN are listed. The observed outcome measures are (a) spatial similarity (η^2^), (b) temporal fluctuation magnitude, and (c) BNC. The coefficients of the estimated ARMA model conform to the following equation:
$$Y_{t}+a_{1}y_{t-1}+a_{2}y_{t-2}+a_{3}y_{t-3}=\;e_{t}+c_{1}e_{t-1}+c_{2}e_{t-2}+c_{3}e_{t-3},$$
where autoregressive (AR) coefficients are listed on top rows and moving-average (MA) coefficients are listed on bottom.(DOCX)

S1 Text Detailed explanation of the autoregressive moving average (ARMA) models. (DOCX)

## References

[1] Biswal B, Yetkin FZ, Haughton VM, Hyde JS. Functional connectivity in the motor cortex of resting human brain using echo-planar MRI. Magn Reson Med. 1995;34: 537–541. 8524021 10.1002/mrm.1910340409

[2] Belegu V, Oudega M, Gary DS, McDonald JW. Restoring function after spinal cord injury: Promoting spontaneous regeneration with stem cells and activity-based therapies. Neurosurg Clin N Am. 2007;18: 143–68, xi 17244561 10.1016/j.nec.2006.10.012

[3] Lorenz DJ, Datta S, Harkema SJ. Longitudinal patterns of functional recovery in patients with incomplete spinal cord injury receiving activity-based rehabilitation. Arch Phys Med Rehabil. 2012;93: 1541–1552. 10.1016/j.apmr.2012.01.027 22920451

[4] Damiano DL. Activity, activity, activity: Rethinking our physical therapy approach to cerebral palsy. Phys Ther. 2006;86: 1534–1540. 17094192 10.2522/ptj.20050397

[5] Martin JH, Chakrabarty S, Friel KM. Harnessing activity-dependent plasticity to repair the damaged corticospinal tract in an animal model of cerebral palsy. Dev Med Child Neurol. 2011;53 Suppl 4: 9–13. 10.1111/j.1469-8749.2011.04055.x 21950387 PMC3187875

[6] Choe AS, Belegu V, Yoshida S, Joel S, Sadowsky CL, Smith SA, et al Extensive neurological recovery from a complete spinal cord injury: A case report and hypothesis on the role of cortical plasticity. Front Hum Neurosci. 2013;7: 290 10.3389/fnhum.2013.00290 23805087 PMC3691521

[7] Helmich RC, Derikx LC, Bakker M, Scheeringa R, Bloem BR, Toni I. Spatial remapping of cortico-striatal connectivity in parkinson's disease. Cereb Cortex. 2010;20: 1175–1186. 10.1093/cercor/bhp178 19710357

[8] Hacker CD, Perlmutter JS, Criswell SR, Ances BM, Snyder AZ. Resting state functional connectivity of the striatum in parkinson's disease. Brain. 2012;135: 3699–3711. 10.1093/brain/aws281 23195207 PMC3525055

[9] Richiardi J, Gschwind M, Simioni S, Annoni JM, Greco B, Hagmann P, et al Classifying minimally disabled multiple sclerosis patients from resting state functional connectivity. Neuroimage. 2012;62: 2021–2033. 10.1016/j.neuroimage.2012.05.078 22677149

[10] Filippi M, Agosta F, Spinelli EG, Rocca MA. Imaging resting state brain function in multiple sclerosis. J Neurol. 2013;260: 1709–1713. 10.1007/s00415-012-6695-z 23052604

[11] Inman CS, James GA, Hamann S, Rajendra JK, Pagnoni G, Butler AJ. Altered resting-state effective connectivity of fronto-parietal motor control systems on the primary motor network following stroke. Neuroimage. 2012;59: 227–237. 10.1016/j.neuroimage.2011.07.083 21839174 PMC3195990

[12] Landman BA, Huang AJ, Gifford A, Vikram DS, Lim IA, Farrell JA, et al Multi-parametric neuroimaging reproducibility: A 3-T resource study. Neuroimage. 2011;54: 2854–2866. 10.1016/j.neuroimage.2010.11.047 21094686 PMC3020263

[13] Stehling MK, Turner R, Mansfield P. Echo-planar imaging: Magnetic resonance imaging in a fraction of a second. Science. 1991;254: 43–50. 1925560 10.1126/science.1925560

[14] Pruessmann KP, Weiger M, Scheidegger MB, Boesiger P. SENSE: Sensitivity encoding for fast MRI. Magn Reson Med. 1999;42: 952–962. 10542355

[15] Friston KJ, Holmes AP, Worsley KJ, Poline JP, Frith CD, Frackowiak RSJ. Statistical parametric maps in functional imaging: A general linear approach. Human brain mapping. 1994;2: 189–210.

[16] Ashburner J, Friston KJ. Unified segmentation. Neuroimage. 2005;26: 839–851. 15955494 10.1016/j.neuroimage.2005.02.018

[17] Egolf E, Kiehl KA, Calhoun VD. Group ICA of fMRI toolbox (GIFT). 2004.

[18] Calhoun VD, Adali T, Pearlson GD, Pekar JJ. A method for making group inferences from functional MRI data using independent component analysis. Hum Brain Mapp. 2001;14: 140–151. 11559959 10.1002/hbm.1048 PMC6871952

[19] Li YO, Adali T, Calhoun VD. Estimating the number of independent components for functional magnetic resonance imaging data. Hum Brain Mapp. 2007;28: 1251–1266. 17274023 10.1002/hbm.20359 PMC6871474

[20] Allen EA, Erhardt EB, Damaraju E, Gruner W, Segall JM, Silva RF, et al A baseline for the multivariate comparison of resting-state networks. Front Syst Neurosci. 2011;5: 2 10.3389/fnsys.2011.00002 21442040 PMC3051178

[21] Allen EA, Erhardt EB, Wei Y, Eichele T, Calhoun VD. Capturing inter-subject variability with group independent component analysis of fMRI data: A simulation study. Neuroimage. 2011;59: 4141–4159. 10.1016/j.neuroimage.2011.10.010 22019879 PMC3690335

[22] Bell AJ, Sejnowski TJ. An information-maximization approach to blind separation and blind deconvolution. Neural Comput. 1995;7: 1129–1159. 7584893 10.1162/neco.1995.7.6.1129

[23] McKeown MJ, Jung TP, Makeig S, Brown G, Kindermann SS, Lee TW, et al Spatially independent activity patterns in functional MRI data during the stroop color-naming task. Proc Natl Acad Sci U S A. 1998;95: 803–810. 9448244 10.1073/pnas.95.3.803 PMC33801

[24] Beckmann CF, DeLuca M, Devlin JT, Smith SM. Investigations into resting-state connectivity using independent component analysis. Philos Trans R Soc Lond B Biol Sci. 2005;360: 1001–1013. 16087444 10.1098/rstb.2005.1634 PMC1854918

[25] Himberg J, Hyvarinen A, Esposito F. Validating the independent components of neuroimaging time series via clustering and visualization. Neuroimage. 2004;22: 1214–1222. 15219593 10.1016/j.neuroimage.2004.03.027

[26] Cohen AL, Fair DA, Dosenbach NU, Miezin FM, Dierker D, Van Essen DC, et al Defining functional areas in individual human brains using resting functional connectivity MRI. Neuroimage. 2008;41: 45–57. 10.1016/j.neuroimage.2008.01.066 18367410 PMC2705206

[27] Kelly C, Uddin LQ, Shehzad Z, Margulies DS, Castellanos FX, Milham MP, et al Broca's region: Linking human brain functional connectivity data and non-human primate tracing anatomy studies. Eur J Neurosci. 2010;32: 383–398. 10.1111/j.1460-9568.2010.07279.x 20662902 PMC3111969

[28] Kim JH, Lee JM, Jo HJ, Kim SH, Lee JH, Kim ST, et al Defining functional SMA and pre-SMA subregions in human MFC using resting state fMRI: Functional connectivity-based parcellation method. Neuroimage. 2010;49: 2375–2386. 10.1016/j.neuroimage.2009.10.016 19837176 PMC2819173

[29] Chen S, Ross TJ, Zhan W, Myers CS, Chuang KS, Heishman SJ, et al Group independent component analysis reveals consistent resting-state networks across multiple sessions. Brain Res. 2008;1239: 141–151. 10.1016/j.brainres.2008.08.028 18789314 PMC2784277

[30] Jafri MJ, Pearlson GD, Stevens M, Calhoun VD. A method for functional network connectivity among spatially independent resting-state components in schizophrenia. Neuroimage. 2008;39: 1666–1681. 18082428 10.1016/j.neuroimage.2007.11.001 PMC3164840

[31] Joel SE, Caffo BS, van Zijl PC, Pekar JJ. On the relationship between seed-based and ICA-based measures of functional connectivity. Magn Reson Med. 2011;66: 644–657. 10.1002/mrm.22818 21394769 PMC3130118

[32] Power JD, Barnes KA, Snyder AZ, Schlaggar BL, Petersen SE. Spurious but systematic correlations in functional connectivity MRI networks arise from subject motion. Neuroimage. 2012;59: 2142–2154. 10.1016/j.neuroimage.2011.10.018 22019881 PMC3254728

[33] Friedman L, Glover GH. Report on a multicenter fMRI quality assurance protocol. J Magn Reson Imaging. 2006;23: 827–839. 16649196 10.1002/jmri.20583

[34] Ahdesmaki M, Lahdesmaki H, Pearson R, Huttunen H, Yli-Harja O. Robust detection of periodic time series measured from biological systems. BMC Bioinformatics. 2005;6: 117 15892890 10.1186/1471-2105-6-117 PMC1168888

[35] Ahdesmaki M, Lahdesmaki H, Gracey A, Shmulevich L, Yli-Harja O. Robust regression for periodicity detection in non-uniformly sampled time-course gene expression data. BMC Bioinformatics. 2007;8: 233 17605777 10.1186/1471-2105-8-233 PMC1934414

[36] Broersen PMT, de Waele S, Bos R. Autoregressive spectral analysis when observations are missing. Automatica. 2004;40: 1495–1504.

[37] Broersen PMT. Automatic spectral analysis with missing data. Digital Signal Processing. 2006;16: 754–766.

[38] Meindl T, Teipel S, Elmouden R, Mueller S, Koch W, Dietrich O, et al Test-retest reproducibility of the default-mode network in healthy individuals. Hum Brain Mapp. 2010;31: 237–246. 10.1002/hbm.20860 19621371 PMC6871144

[39] Li Z, Kadivar A, Pluta J, Dunlop J, Wang Z. Test-retest stability analysis of resting brain activity revealed by blood oxygen level-dependent functional MRI. J Magn Reson Imaging. 2012;36: 344–354. 10.1002/jmri.23670 22535702 PMC3399952

[40] Wisner KM, Atluri G, Lim KO, Macdonald AW 3rd. Neurometrics of intrinsic connectivity networks at rest using fMRI: Retest reliability and cross-validation using a meta-level method. Neuroimage. 2013;76: 236–251. 10.1016/j.neuroimage.2013.02.066 23507379 PMC8753639

[41] Damoiseaux JS, Rombouts SA, Barkhof F, Scheltens P, Stam CJ, Smith SM, et al Consistent resting-state networks across healthy subjects. Proc Natl Acad Sci U S A. 2006;103: 13848–13853. 16945915 10.1073/pnas.0601417103 PMC1564249

[42] Guo CC, Kurth F, Zhou J, Mayer EA, Eickhoff SB, Kramer JH, et al One-year test-retest reliability of intrinsic connectivity network fMRI in older adults. Neuroimage. 2012;61: 1471–1483. 10.1016/j.neuroimage.2012.03.027 22446491 PMC4226138

[43] Raichle ME, MacLeod AM, Snyder AZ, Powers WJ, Gusnard DA, Shulman GL. A default mode of brain function. Proc Natl Acad Sci U S A. 2001;98: 676–682. 11209064 10.1073/pnas.98.2.676 PMC14647

[44] Weiland BJ, Sabbineni A, Calhoun VD, Welsh RC, Hutchison KE. Reduced executive and default network functional connectivity in cigarette smokers. Hum Brain Mapp. 2014. 10.1002/hbm.22672 PMC4978515 25346448

[45] Seeley WW, Menon V, Schatzberg AF, Keller J, Glover GH, Kenna H, et al Dissociable intrinsic connectivity networks for salience processing and executive control. J Neurosci. 2007;27: 2349–2356. 17329432 10.1523/JNEUROSCI.5587-06.2007 PMC2680293

[46] Segall JM, Allen EA, Jung RE, Erhardt EB, Arja SK, Kiehl K, et al Correspondence between structure and function in the human brain at rest. Front Neuroinform. 2012;6: 10 10.3389/fninf.2012.00010 22470337 PMC3313067

[47] Kosslyn SM. Mental images and the brain. Cogn Neuropsychol. 2005;22: 333–347. 10.1080/02643290442000130 21038254

[48] Miller MI, Beg MF, Ceritoglu C, Stark C. Increasing the power of functional maps of the medial temporal lobe by using large deformation diffeomorphic metric mapping. Proc Natl Acad Sci U S A. 2005;102: 9685–9690. 15980148 10.1073/pnas.0503892102 PMC1172268

[49] Oishi K, Faria A, Jiang H, Li X, Akhter K, Zhang J, et al Atlas-based whole brain white matter analysis using large deformation diffeomorphic metric mapping: Application to normal elderly and alzheimer's disease participants. Neuroimage. 2009;46: 486–499. 19385016 10.1016/j.neuroimage.2009.01.002 PMC2885858

[50] Yang Z, Zuo XN, Wang P, Li Z, LaConte SM, Bandettini PA, et al Generalized RAICAR: Discover homogeneous subject (sub)groups by reproducibility of their intrinsic connectivity networks. Neuroimage. 2012;63: 403–414. 10.1016/j.neuroimage.2012.06.060 22789741

[51] Krmpotich TD, Tregellas JR, Thompson LL, Banich MT, Klenk AM, Tanabe JL. Resting-state activity in the left executive control network is associated with behavioral approach and is increased in substance dependence. Drug Alcohol Depend. 2013;129: 1–7. 10.1016/j.drugalcdep.2013.01.021 23428318 PMC3618865

[52] Samann PG, Wehrle R, Hoehn D, Spoormaker VI, Peters H, Tully C, et al Development of the brain's default mode network from wakefulness to slow wave sleep. Cereb Cortex. 2011;21: 2082–2093. 10.1093/cercor/bhq295 21330468

[53] Mazoyer B, Zago L, Mellet E, Bricogne S, Etard O, Houde O, et al Cortical networks for working memory and executive functions sustain the conscious resting state in man. Brain Res Bull. 2001;54: 287–298. 11287133 10.1016/s0361-9230(00)00437-8

[54] Agosta F, Pievani M, Geroldi C, Copetti M, Frisoni GB, Filippi M. Resting state fMRI in alzheimer's disease: Beyond the default mode network. Neurobiol Aging. 2012;33: 1564–1578. 10.1016/j.neurobiolaging.2011.06.007 21813210

[55] Ge Y, Grossman RI, Babb JS, Rabin ML, Mannon LJ, Kolson DL. Age-related total gray matter and white matter changes in normal adult brain. part I: Volumetric MR imaging analysis. AJNR Am J Neuroradiol. 2002;23: 1327–1333. 12223373 PMC7976241

[56] Pfefferbaum A, Mathalon DH, Sullivan EV, Rawles JM, Zipursky RB, Lim KO. A quantitative magnetic resonance imaging study of changes in brain morphology from infancy to late adulthood. Arch Neurol. 1994;51: 874–887. 8080387 10.1001/archneur.1994.00540210046012

[57] Courchesne E, Chisum HJ, Townsend J, Cowles A, Covington J, Egaas B, et al Normal brain development and aging: Quantitative analysis at in vivo MR imaging in healthy volunteers. Radiology. 2000;216: 672–682. 10966694 10.1148/radiology.216.3.r00au37672

[58] Webb SJ, Monk CS, Nelson CA. Mechanisms of postnatal neurobiological development: Implications for human development. Dev Neuropsychol. 2001;19: 147–171. 11530973 10.1207/S15326942DN1902_2

[59] Jones CK, Calabresi PA, Barker PB, van Zijl PC. Weekly scanning of a normal control over four years. 22nd Scientific Meeting and Exhibition, International Society of Magnetic Resonance in Medicine 2014, Milan Italy. 2014: 1775.

[60] Geerligs L, Renken RJ, Saliasi E, Maurits NM, Lorist MM. A brain-wide study of age-related changes in functional connectivity. Cereb Cortex. 2014. 10.1093/cercor/bhu012 24532319

[61] Vogel AC, Power JD, Petersen SE, Schlaggar BL. Development of the brain's functional network architecture. Neuropsychol Rev. 2010;20: 362–375. 10.1007/s11065-010-9145-7 20976563 PMC3811138

[62] Sato JR, Salum GA, Gadelha A, Picon FA, Pan PM, Vieira G, et al Age effects on the default mode and control networks in typically developing children. J Psychiatr Res. 2014;58C: 89–95. 10.1016/j.jpsychires.2014.07.004 25085608

[63] Koch W, Teipel S, Mueller S, Buerger K, Bokde AL, Hampel H, et al Effects of aging on default mode network activity in resting state fMRI: Does the method of analysis matter? Neuroimage. 2010;51: 280–287. 10.1016/j.neuroimage.2009.12.008 20004726

[64] Damoiseaux JS, Beckmann CF, Arigita EJ, Barkhof F, Scheltens P, Stam CJ, et al Reduced resting-state brain activity in the "default network" in normal aging. Cereb Cortex. 2008;18: 1856–1864. 18063564 10.1093/cercor/bhm207

[65] Andrews-Hanna JR, Snyder AZ, Vincent JL, Lustig C, Head D, Raichle ME, et al Disruption of large-scale brain systems in advanced aging. Neuron. 2007;56: 924–935. 18054866 10.1016/j.neuron.2007.10.038 PMC2709284

[66] Fair DA, Cohen AL, Dosenbach NU, Church JA, Miezin FM, Barch DM, et al The maturing architecture of the brain's default network. Proc Natl Acad Sci U S A. 2008;105: 4028–4032. 10.1073/pnas.0800376105 18322013 PMC2268790

[67] Karunanayaka PR, Holland SK, Schmithorst VJ, Solodkin A, Chen EE, Szaflarski JP, et al Age-related connectivity changes in fMRI data from children listening to stories. Neuroimage. 2007;34: 349–360. 17064940 10.1016/j.neuroimage.2006.08.028

[68] Szaflarski JP, Holland SK, Schmithorst VJ, Byars AW. fMRI study of language lateralization in children and adults. Hum Brain Mapp. 2006;27: 202–212. 16035047 10.1002/hbm.20177 PMC1464420

[69] Kramer AF, Bherer L, Colcombe SJ, Dong W, Greenough WT. Environmental influences on cognitive and brain plasticity during aging. J Gerontol A Biol Sci Med Sci. 2004;59: M940–57. 15472160 10.1093/gerona/59.9.m940

[70] Pesonen AK, Eriksson JG, Heinonen K, Kajantie E, Tuovinen S, Alastalo H, et al Cognitive ability and decline after early life stress exposure. Neurobiol Aging. 2013;34: 1674–1679. 10.1016/j.neurobiolaging.2012.12.012 23337341

[71] Yamada H, Yamamoto MT. Association between circadian clock genes and diapause incidence in drosophila triauraria. PLoS One. 2011;6: e27493 10.1371/journal.pone.0027493 22164210 PMC3229484

[72] Merrow M, Spoelstra K, Roenneberg T. The circadian cycle: Daily rhythms from behaviour to genes. EMBO Rep. 2005;6: 930–935. 16222241 10.1038/sj.embor.7400541 PMC1369194

[73] Paredes SD, Marchena AM, Bejarano I, Espino J, Barriga C, Rial RV, et al Melatonin and tryptophan affect the activity-rest rhythm, core and peripheral temperatures, and interleukin levels in the ringdove: Changes with age. J Gerontol A Biol Sci Med Sci. 2009;64: 340–350. 10.1093/gerona/gln054 19211547 PMC2654999

[74] Schmidt C, Collette F, Cajochen C, Peigneux P. A time to think: Circadian rhythms in human cognition. Cogn Neuropsychol. 2007;24: 755–789. 18066734 10.1080/02643290701754158

[75] Kent BA. Synchronizing an aging brain: Can entraining circadian clocks by food slow alzheimer's disease? Front Aging Neurosci. 2014;6: 234 10.3389/fnagi.2014.00234 25225484 PMC4150207

[76] Anderson JA, Campbell KL, Amer T, Grady CL, Hasher L. Timing is everything: Age differences in the cognitive control network are modulated by time of day. Psychol Aging. 2014;29: 648–657. 10.1037/a0037243 24999661 PMC4898963

[77] Blautzik J, Vetter C, Peres I, Gutyrchik E, Keeser D, Berman A, et al Classifying fMRI-derived resting-state connectivity patterns according to their daily rhythmicity. Neuroimage. 2013;71: 298–306. 10.1016/j.neuroimage.2012.08.010 22906784

[78] Blautzik J, Vetter C, Schneider A, Gutyrchik E, Reinisch V, Keeser D, et al Dysregulated daily rhythmicity of neuronal resting-state networks in MCI patients. Chronobiol Int. 2014: 1–10. 10.3109/07420528.2014.944618 25099642

[79] Hodkinson DJ, O'Daly O, Zunszain PA, Pariante CM, Lazurenko V, Zelaya FO, et al Circadian and homeostatic modulation of functional connectivity and regional cerebral blood flow in humans under normal entrained conditions. J Cereb Blood Flow Metab. 2014;34: 1493–1499. 10.1038/jcbfm.2014.109 24938404 PMC4158665

[80] Hjelmervik H, Hausmann M, Osnes B, Westerhausen R, Specht K. Resting states are resting traits—an FMRI study of sex differences and menstrual cycle effects in resting state cognitive control networks. PLoS One. 2014;9: e103492 10.1371/journal.pone.0103492 25057823 PMC4110030

[81] Sandler RA, Song D, Hampson RE, Deadwyler SA, Berger TW, Marmarelis VZ. Model-based asessment of an in-vivo predictive relationship from CA1 to CA3 in the rodent hippocampus. J Comput Neurosci. 2014. 10.1007/s10827-014-0530-8 PMC4297547 25260381

[82] Stanculescu I, Williams CK, Freer Y. Autoregressive hidden markov models for the early detection of neonatal sepsis. IEEE J Biomed Health Inform. 2014;18: 1560–1570. 10.1109/JBHI.2013.2294692 25192568

[83] Shehzad Z, Kelly AM, Reiss PT, Gee DG, Gotimer K, Uddin LQ, et al The resting brain: Unconstrained yet reliable. Cereb Cortex. 2009;19: 2209–2229. 10.1093/cercor/bhn256 19221144 PMC3896030

[84] Schmithorst VJ, Holland SK. Comparison of three methods for generating group statistical inferences from independent component analysis of functional magnetic resonance imaging data. J Magn Reson Imaging. 2004;19: 365–368. 14994306 10.1002/jmri.20009 PMC2265794

[85] Amann M, Hirsch JG, Gass A. A serial functional connectivity MRI study in healthy individuals assessing the variability of connectivity measures: Reduced interhemispheric connectivity in the motor network during continuous performance. Magn Reson Imaging. 2009;27: 1347–1359. 10.1016/j.mri.2009.05.016 19559557

[86] Anderson JS, Ferguson MA, Lopez-Larson M, Yurgelun-Todd D. Reproducibility of single-subject functional connectivity measurements. AJNR Am J Neuroradiol. 2011;32: 548–555. 10.3174/ajnr.A2330 21273356 PMC3205089

[87] Chou YH, Panych LP, Dickey CC, Petrella JR, Chen NK. Investigation of long-term reproducibility of intrinsic connectivity network mapping: A resting-state fMRI study. AJNR Am J Neuroradiol. 2012;33: 833–838. 10.3174/ajnr.A2894 22268094 PMC3584561

[88] Seibert TM, Majid DS, Aron AR, Corey-Bloom J, Brewer JB. Stability of resting fMRI interregional correlations analyzed in subject-native space: A one-year longitudinal study in healthy adults and premanifest huntington's disease. Neuroimage. 2012;59: 2452–2463. 10.1016/j.neuroimage.2011.08.105 21945695 PMC3254727

[89] Zuo XN, Kelly C, Adelstein JS, Klein DF, Castellanos FX, Milham MP. Reliable intrinsic connectivity networks: Test-retest evaluation using ICA and dual regression approach. Neuroimage. 2010;49: 2163–2177. 10.1016/j.neuroimage.2009.10.080 19896537 PMC2877508

