# Supplementary figures and images for: Reproducibility and Temporal Structure in Weekly Resting-State fMRI over a Period of 3.5 Years
Source: PLoS One. 2015 Oct 30;10(10):e0140134. doi: 10.1371/journal.pone.0140134 (PMC4627782; doi:10.1371/journal.pone.0140134)

S1 Figure. Preprocessing and group independent component analysis (GICA) flowchart


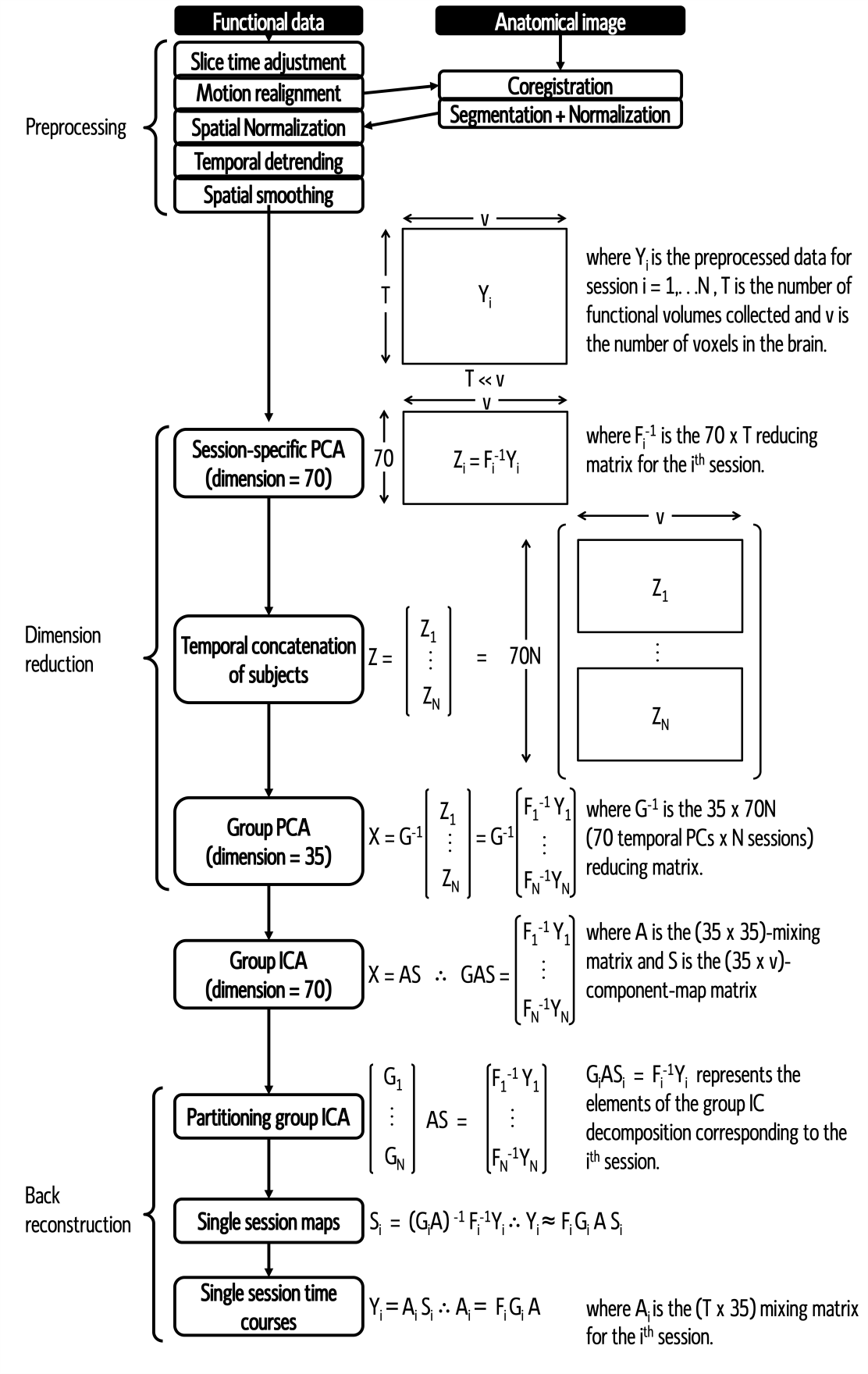

Supplement: S1 Fig — (DOCX) [file pone.0140134.s001.docx]
