# Supplementary material for: Reproducibility and Temporal Structure in Weekly Resting-State fMRI over a Period of 3.5 Years
Source: PLoS One. 2015 Oct 30;10(10):e0140134. doi: 10.1371/journal.pone.0140134 (PMC4627782; doi:10.1371/journal.pone.0140134)
Supplement: S2 Fig — Spatial similarity of each session’s RSN spatial map to the corresponding group mean map, measured using eta-squared (η2), for single-subject (a) and multi-participant (b) datasets, is visualized using box plots (end of boxes: quartiles, bar within boxes: median, small dots: outliers). In (b), for each RSN, the mean η2 of the single-subject dataset is overlaid as a large gray circle. (DOCX) [file pone.0140134.s002.docx]

S2 Figure. Reproducibility of resting state network (RSN) spatial maps, visualized using boxplots.

Spatial similarity of each session’s RSN spatial map to the corresponding group mean map, measured using eta-squared (η^2^), for single-subject (a) and multi-participant (b) datasets, is visualized using box plots (end of boxes: quartiles, bar within boxes: median, small dots: outliers). In (b), for each RSN, the mean η^2^ of the single-subject dataset is overlaid as a large gray circle.
