# Supplementary material for: Reproducibility and Temporal Structure in Weekly Resting-State fMRI over a Period of 3.5 Years
Source: PLoS One. 2015 Oct 30;10(10):e0140134. doi: 10.1371/journal.pone.0140134 (PMC4627782; doi:10.1371/journal.pone.0140134)
Supplement: S3 Fig — Blood oxygenation level dependent (BOLD) signal fluctuation magnitude for each session’s RSN time courses, calculated as root-mean-squared (RMS) % BOLD for single-subject (a) and multi-participant (b) datasets, is visualized using boxplots. In (b), for each RSN, the mean RMS % BOLD value for the single-subject dataset is overlaid as a large gray circle. (DOCX) [file pone.0140134.s003.docx]

S3 Figure. Reproducibility of RSN signal temporal fluctuation magnitude, visualized using boxplots.

Blood oxygenation level dependent (BOLD) signal fluctuation magnitude for each session’s RSN time courses, calculated as root-mean-squared (RMS) % BOLD for single-subject (a) and multi-participant (b) datasets, is visualized using boxplots. In (b), for each RSN, the mean RMS % BOLD value for the single-subject dataset is overlaid as a large gray circle.
