# Supplementary material for: Reproducibility and Temporal Structure in Weekly Resting-State fMRI over a Period of 3.5 Years
Source: PLoS One. 2015 Oct 30;10(10):e0140134. doi: 10.1371/journal.pone.0140134 (PMC4627782; doi:10.1371/journal.pone.0140134)
Supplement: S1 Table — A total of 158 scans were acquired over the period of 185 weeks. (DOCX) [file pone.0140134.s004.docx]

S1 Table. Acquisition dates of the 158 resting state functional MRI (rs-fMRI) scans.

| **2009** | | | | | | | | |
| --- | --- | --- | --- | --- | --- | --- | --- | --- |
| Dec - 07 | Dec - 14 | Dec - 21 | Dec - 31^¶^ |  |  |  |  |  |
| **2010** | | | | | | | | |
| Jan - 06 | Jan - 14 | Jan - 21 | Jan - 28 | Feb - 04 | Feb - 12 | Feb - 18 | Feb - 26 | Mar - 04 |
| Mar - 11 | Mar - 19 | Mar - 25 | Apr - 01^¶^ | Apr - 07 | Apr - 15 | Apr - 22 | Apr - 27 | May - 06^¶^ |
| May - 13 | May - 20 | May - 27 | Jun - 07 | Jun - 10 | Jun - 17 | Jun - 24 | Jul - 01 | Jul - 09 |
| Jul - 16^¶^ | Jul - 22 | Jul - 29 | Aug - 05 | Aug - 12 | Aug - 19 | Aug - 26 | Sep - 02 | Sep - 09 |
| Sep - 16 | Sep - 23 | Sep - 30^¶^ | Oct - 06 | Oct - 13 | Oct - 21 | Oct - 27 | Nov - 04 | Nov - 11 |
| Nov - 18 | Nov - 24 | Dec - 02 | Dec - 09 | Dec - 16 | Dec - 22 | Dec-30^¶^ |  |  |
| **2011** | | | | | | | | |
| Jan - 06 | Jan - 12 | Jan - 20 | Jan - 27^¶^ | Feb - 03 | Feb - 10 | Feb - 17 | Feb - 24 | Mar - 04 |
| Mar - 10 | Mar - 17 | Mar - 24 | Mar - 31 | Apr - 08 | Apr - 14 | Apr - 21 | Apr - 28 | May - 05 |
| May - 12^¶^ | May - 19 | May - 26 | Jun - 02 | Jun - 10 | Jun - 14 | Jun - 23 | Jun - 30 | Jul - 07 |
| Jul – 14 | Jul - 21^¶^ | Jul – 28 | Aug - 04^¶^ | Aug - 11 | Aug - 18 | Aug - 25^¶^ | Sep - 01 | Sep - 09 |
| Sep - 15 | Sep - 22^¶^ | Sep - 29 | Oct - 06 | Oct - 12 | Oct - 20 | Oct - 27 | Nov - 03 | Nov - 11 |
| Nov - 17 | Nov - 23 | Dec - 1^¶^ | Dec - 08 | Dec - 15 | Dec - 22^¶^ |  |  |  |
| **2012** | | | | | | | | |
| Jan - 03 | Jan - 05 | Jan - 12 | Jan - 19 | Jan - 26 | Feb - 02 | Feb - 09 | Feb - 16 | Feb - 23 |
| Mar - 02 | Mar - 08 | Mar - 15 | Mar - 23 | Mar - 28 | Apr - 06 | Apr - 12 | Apr - 20 | Apr - 25 |
| May - 03 | May - 10 | May - 17 | May - 24 | May - 31 | Jun - 07 | Jun - 14 | Jun - 21 | Jun - 28^¶^ |
| Jul - 5^¶^ | Jul - 12 | Jul - 19^¶^ | Jul - 26 | Aug - 03 | Aug - 13 | Aug - 15 | Aug - 23 | Aug - 31 |
| Sep - 6^¶^ | Sep - 13^¶^ | Sep - 19 | Sep - 27^¶^ | Oct - 03 | Oct - 12 | Oct - 18 | Oct - 23 | Nov - 01 |
| Nov - 08 | Nov - 15 | Nov - 22^¶^ | Nov - 29^¶^ | Dec - 06 | Dec - 14 | Dec - 20^¶^ | Dec - 27^¶^ |  |
| **2013** | | | | | | | | |
| Jan - 03 | Jan - 10 | Jan - 18 | Jan - 23 | Jan - 31 | Feb - 07 | Feb - 14 | Feb - 21 | Feb - 28 |
| Mar - 07 | Mar - 14 | Mar - 21 | Mar - 28 | Apr - 03 | Apr - 11 | Apr - 18^¶^ | Apr - 25^¶^ | May - 02 |
| May - 10 | May - 16 | May - 23 | May - 30^¶^ | Jun - 06 | Jun - 13 | Jun - 20 |  |  |

^¶^ Weeks of missing scans

A total of 158 scans were acquired over the period of 185 weeks.
