# Supplementary material for: Reproducibility and Temporal Structure in Weekly Resting-State fMRI over a Period of 3.5 Years
Source: PLoS One. 2015 Oct 30;10(10):e0140134. doi: 10.1371/journal.pone.0140134 (PMC4627782; doi:10.1371/journal.pone.0140134)
Supplement: S2 Table — The mean and standard deviation (SD) values for all resting state network (RSN) pairs are shown for the single- and multi-participant datasets. (Aud: auditory, Smot: seonsorimotor, Vis: visual, DMN: default mode network, Attn: attention, Exec: executive, Sal: salience, Cb: cerebellar, ven: ventral, dor: dorsal, R: right, L: left). (DOCX) [file pone.0140134.s005.docx]

S2 Table. Reproducibility of single- and multi-participant between-network connectivity (BNC) measurements

|  | BNC | | | |  | BNC | | | |
| --- | --- | --- | --- | --- | --- | --- | --- | --- | --- |
| RSN pairs | Single-subject | | Multi-participant | | RSN pairs | Single-subject | | Multi-participant | |
|  | Mean | SD | Mean | SD |  | Mean | SD | Mean | SD |
| Aud / Smot-dor | 0.391 | 0.226 | 0.357 | 0.200 | DMN-c / Cb | 0.219 | 0.125 | 0.342 | 0.174 |
| Aud / Sal | 0.279 | 0.129 | 0.334 | 0.196 | DMN-c / Attn-ven | 0.134 | 0.250 | 0.218 | 0.209 |
| Aud / Vis-b | 0.482 | 0.173 | 0.309 | 0.180 | DMN-c / DMN-a | 0.340 | 0.145 | 0.214 | 0.227 |
| Aud / DMN-c | 0.133 | 0.249 | 0.167 | 0.233 | DMN-c / Exec-L | 0.158 | 0.147 | 0.197 | 0.143 |
| Aud / Cb | 0.360 | 0.123 | 0.323 | 0.201 | DMN-c / Attn-dor | -0.065 | 0.241 | 0.076 | 0.281 |
| Aud / Attn-ven | 0.458 | 0.169 | 0.265 | 0.207 | DMN-c / DMN-b | 0.276 | 0.198 | 0.411 | 0.208 |
| Aud / DMN-a | 0.469 | 0.168 | 0.248 | 0.181 | DMN-c / Smot-ven | 0.194 | 0.287 | 0.241 | 0.283 |
| Aud / Exec-L | -0.033 | 0.153 | 0.012 | 0.228 | DMN-c / Vis-a | 0.226 | 0.241 | 0.275 | 0.224 |
| Aud / Attn-dor | 0.505 | 0.164 | 0.402 | 0.179 | DMN-c / Exec-R | 0.306 | 0.151 | 0.387 | 0.162 |
| Aud / DMN-b | 0.527 | 0.182 | 0.345 | 0.247 | Cb / Attn-ven | 0.124 | 0.106 | 0.128 | 0.228 |
| Aud / Smot-ven | 0.660 | 0.126 | 0.567 | 0.187 | Cb / DMN-a | 0.196 | 0.124 | 0.124 | 0.179 |
| Aud / Vis-a | 0.538 | 0.171 | 0.306 | 0.160 | Cb / Exec-L | 0.076 | 0.103 | 0.140 | 0.147 |
| Aud / Exec-R | 0.174 | 0.174 | 0.184 | 0.214 | Cb / Attn-dor | 0.237 | 0.139 | 0.232 | 0.222 |
| Smot-dor / Sal | 0.334 | 0.144 | 0.223 | 0.263 | Cb / DMN-b | 0.338 | 0.110 | 0.325 | 0.209 |
| Smot-dor / Vis-b | 0.650 | 0.135 | 0.445 | 0.223 | Cb / Smot-ven | 0.207 | 0.103 | 0.177 | 0.188 |
| Smot-dor / DMN-c | 0.272 | 0.285 | 0.341 | 0.231 | Cb / Vis-a | 0.231 | 0.101 | 0.177 | 0.181 |
| Smot-dor / Cb | 0.121 | 0.129 | 0.234 | 0.225 | Cb / Exec-R | 0.186 | 0.127 | 0.151 | 0.159 |
| Smot-dor / Attn-ven | 0.382 | 0.228 | 0.296 | 0.232 | Attn-ven / DMN-a | 0.502 | 0.172 | 0.395 | 0.154 |
| Smot-dor / DMN-a | 0.241 | 0.196 | 0.169 | 0.231 | Attn-ven / Exec-L | 0.103 | 0.175 | 0.139 | 0.210 |
| Smot-dor / Exec-L | 0.066 | 0.162 | 0.120 | 0.265 | Attn-ven / Attn-dor | 0.378 | 0.208 | 0.146 | 0.285 |
| Smot-dor / Attn-dor | 0.281 | 0.233 | 0.396 | 0.235 | Attn-ven / DMN-b | 0.292 | 0.240 | 0.222 | 0.233 |
| Smot-dor / DMN-b | 0.506 | 0.192 | 0.364 | 0.214 | Attn-ven / Smot-ven | 0.501 | 0.215 | 0.345 | 0.271 |
| Smot-dor / Smot-ven | 0.551 | 0.204 | 0.554 | 0.152 | Attn-ven / Vis-a | 0.491 | 0.211 | 0.201 | 0.242 |
| Smot-dor / Vis-a | 0.538 | 0.212 | 0.488 | 0.182 | Attn-ven / Exec-R | 0.328 | 0.163 | 0.335 | 0.200 |
| Smot-dor / Exec-R | 0.107 | 0.163 | 0.134 | 0.244 | DMN-a / Exec-L | -0.078 | 0.180 | 0.087 | 0.175 |
| Sal / Vis-b | 0.204 | 0.119 | 0.139 | 0.234 | DMN-a / Attn-dor | 0.239 | 0.230 | 0.103 | 0.175 |
| Sal / DMN-c | -0.045 | 0.239 | 0.076 | 0.222 | DMN-a / DMN-b | 0.276 | 0.196 | 0.191 | 0.180 |
| Sal / Cb | -0.038 | 0.122 | 0.080 | 0.149 | DMN-a / Smot-ven | 0.489 | 0.158 | 0.259 | 0.268 |
| Sal / Attn-ven | -0.004 | 0.173 | -0.075 | 0.282 | DMN-a / Vis-a | 0.418 | 0.158 | 0.217 | 0.174 |
| Sal / DMN-a | -0.133 | 0.178 | -0.104 | 0.230 | DMN-a / Exec-R | 0.200 | 0.199 | 0.200 | 0.204 |
| Sal / Exec-L | 0.183 | 0.142 | 0.244 | 0.134 | Exec-L / Attn-dor | 0.256 | 0.128 | 0.255 | 0.187 |
| Sal / Attn-dor | 0.307 | 0.151 | 0.306 | 0.234 | Exec-L / DMN-b | 0.121 | 0.163 | 0.209 | 0.157 |
| Sal / DMN-b | 0.233 | 0.140 | 0.222 | 0.217 | Exec-L / Smot-ven | 0.066 | 0.168 | 0.106 | 0.225 |
| Sal / Smot-ven | 0.287 | 0.141 | 0.297 | 0.227 | Exec-L / Vis-a | 0.032 | 0.205 | 0.066 | 0.283 |
| Sal / Vis-a | 0.140 | 0.151 | 0.083 | 0.246 | Exec-L / Exec-R | 0.599 | 0.082 | 0.509 | 0.152 |
| Sal / Exec-R | 0.106 | 0.134 | 0.146 | 0.209 | Attn-dor / DMN-b | 0.371 | 0.184 | 0.350 | 0.218 |
| Vis-b / DMN-c | 0.224 | 0.249 | 0.159 | 0.247 | Attn-dor / Smot-ven | 0.472 | 0.182 | 0.374 | 0.230 |
| Vis-b / Cb | 0.296 | 0.114 | 0.254 | 0.187 | Attn-dor / Vis-a | 0.414 | 0.211 | 0.359 | 0.210 |
| Vis-b / Attn-ven | 0.456 | 0.199 | 0.321 | 0.224 | Attn-dor / Exec-R | 0.294 | 0.128 | 0.212 | 0.204 |
| Vis-b / DMN-a | 0.326 | 0.205 | 0.229 | 0.195 | DMN-b / Smot-ven | 0.385 | 0.217 | 0.270 | 0.231 |
| Vis-b / Exec-L | 0.189 | 0.153 | 0.171 | 0.269 | DMN-b / Vis-a | 0.509 | 0.238 | 0.308 | 0.171 |
| Vis-b / Attn-dor | 0.358 | 0.233 | 0.412 | 0.230 | DMN-b / Exec-R | 0.256 | 0.153 | 0.345 | 0.165 |
| Vis-b / DMN-b | 0.554 | 0.208 | 0.398 | 0.215 | Smot-ven / Vis-a | 0.593 | 0.167 | 0.453 | 0.163 |
| Vis-b / Smot-ven | 0.479 | 0.183 | 0.361 | 0.222 | Smot-ven / Exec-R | 0.172 | 0.168 | 0.143 | 0.242 |
| Vis-b / Vis-a | 0.636 | 0.166 | 0.606 | 0.191 | Vis-a / Exec-R | 0.138 | 0.168 | 0.121 | 0.217 |
| Vis-b / Exec-R | 0.244 | 0.173 | 0.196 | 0.251 |  |  |  |  |  |

The mean and standard deviation (SD) values for all resting state network (RSN) pairs are shown for the single- and multi-participant datasets. (Aud: auditory, Smot: seonsorimotor, Vis: visual, DMN: default mode network, Attn: attention, Exec: executive, Sal: salience, Cb: cerebellar, ven: ventral, dor: dorsal, R: right, L: left)
