# Supplementary material for: Reproducibility and Temporal Structure in Weekly Resting-State fMRI over a Period of 3.5 Years
Source: PLoS One. 2015 Oct 30;10(10):e0140134. doi: 10.1371/journal.pone.0140134 (PMC4627782; doi:10.1371/journal.pone.0140134)
Supplement: S3 Table — Properties of the estimated ARMA models for three outcome measures of each RSN are listed. The observed outcome measures are (a) spatial similarity (η2), (b) temporal fluctuation magnitude, and (c) BNC. The coefficients of the estimated ARMA model conform to the following equation: Yt+a1yt−1+a2yt−2+a3yt−3= et+c1et−1+c2et−2+c3et−3, where autoregressive (AR) coefficients are listed on top rows and moving-average (MA) coefficients are listed on bottom. (DOCX) [file pone.0140134.s006.docx]

S3 Table. Estimated autoregressive moving average (ARMA) models of each RSNs, for three rs-fMRI outcome measures – η^2^, temporal signal fluctuation, and BNC

| (a) Eta-squared (η^2^) | | | | | | | |
| --- | --- | --- | --- | --- | --- | --- | --- |
| RSN | Estimated ARMA model | Portmanteau p-val | Pct variance (%) | Estimated ARMA Coefficient (AR coeff: top, MA coeff: bottom) | | | |
| Aud | AR(1) | 0.92 | 17.92 | 1.00 | -0.22 |  |  |
|  |  |  |  |  |  |  |  |
| Smot-ven | AR(3) | 0.53 | 49.77 | 1.00 | -0.16 | -0.17 | -0.22 |
|  |  |  |  |  |  |  |  |
| Smot-dor | AR(3) | 0.44 | 42.12 | 1.00 | -0.15 | -0.21 | -0.19 |
|  |  |  |  |  |  |  |  |
| Vis-a | AR(2) | 0.42 | 33.42 | 1.00 | -0.19 | -0.29 |  |
|  |  |  |  |  |  |  |  |
| Vis-b | AR(2) | 0.85 | 37.42 | 1.00 | -0.22 | -0.30 |  |
|  |  |  |  |  |  |  |  |
| DMN-a | ARMA(2, 1) | 0.12 | 38.98 | 1.00 | -0.69 | -0.19 |  |
|  |  |  |  | 1.00 | -0.57 |  |  |
| DMN-b | AR(3) | 0.90 | 42.17 | 1.00 | -0.16 | -0.14 | -0.17 |
|  |  |  |  |  |  |  |  |
| Attn-dor | AR(3) | 0.77 | 36.34 | 1.00 | -0.08 | -0.16 | -0.22 |
|  |  |  |  |  |  |  |  |
| Exec-R | AR(2) | 0.18 | 39.10 | 1.00 | -0.17 | -0.22 |  |
|  |  |  |  |  |  |  |  |
| Exec-L | AR(1) | 0.49 | 15.77 | 1.00 | -0.16 |  |  |
|  |  |  |  |  |  |  |  |
| Sal | AR(1) | 0.48 | 14.92 | 1.00 | -0.26 |  |  |
|  |  |  |  |  |  |  |  |
| Cb | AR(2) | 0.49 | 37.37 | 1.00 | -0.25 | -0.29 |  |
|  |  |  |  |  |  |  |  |
| (b) Temporal fluctuation magnitude | | | | | | | |
| RSN | Estimated ARMA model | Portmanteau p-val | Pct variance (%) | Estimated ARMA Coefficient (AR coeff: top, MA coeff: bottom) | | | |
| Aud | ARMA(0, 1) | 0.75 | 24.00 |  |  |  |  |
|  |  |  |  | 1.00 | 0.22 |  |  |
| Vis-a | AR(2) | 0.74 | 29.76 | 1.00 | -0.19 | -0.20 |  |
|  |  |  |  |  |  |  |  |
| Sal | AR(1) | 0.90 | 11.46 | 1.00 | -0.15 |  |  |
|  |  |  |  |  |  |  |  |
| (c) Between-network connectivity | | | | | | | |
| RSN | Estimated ARMA model | Portmanteau p-val | Pct variance (%) | Estimated ARMA Coefficient (AR coeff: top, MA coeff: bottom) | | | |
| Aud / Smot-dor | AR(1) | 0.77 | 15.98 | 1.00 | -0.18 |  |  |
|  |  |  |  |  |  |  |  |
| Aud / DMN-a | AR(1) | 0.43 | 20.16 | 1.00 | -0.23 |  |  |
|  |  |  |  |  |  |  |  |
| Aud / DMN-b | AR(1) | 0.20 | 17.05 | 1.00 | -0.26 |  |  |
|  |  |  |  |  |  |  |  |
| Aud / DMN-c | AR(3) | 0.34 | 39.62 | 1.00 | -0.08 | -0.09 | -0.21 |
|  |  |  |  |  |  |  |  |
| Aud / Attn-dor | AR(1) | 0.88 | 15.25 | 1.00 | -0.18 |  |  |
|  |  |  |  |  |  |  |  |
| Smot-ven / | AR(1) | 0.54 | 17.22 | 1.00 | -0.21 |  |  |
| Smot-dor |  |  |  |  |  |  |  |
| Smot-ven / | AR(1) | 0.48 | 19.98 | 1.00 | -0.28 |  |  |
| DMN-b |  |  |  |  |  |  |  |
| Smot-ven / | AR(3) | 0.41 | 38.23 | 1.00 | -0.12 | -0.12 | -0.20 |
| DMN-c |  |  |  |  |  |  |  |
| Smot-dor / | AR(1) | 0.41 | 19.71 | 1.00 | -0.26 |  |  |
| DMN-b |  |  |  |  |  |  |  |
| Smot-dor / | ARMA(0, 3) | 0.31 | 38.94 |  |  |  |  |
| DMN-c |  |  |  | 1.00 | 0.22 | 0.25 | 0.29 |
| Smot-dor / | AR(1) | 0.85 | 16.11 | 1.00 | -0.18 |  |  |
| Attn-dor |  |  |  |  |  |  |  |
| Vis-a / | AR(2) | 0.16 | 32.19 | 1.00 | -0.21 | -0.15 |  |
| DMN-b |  |  |  |  |  |  |  |
| Vis-a / | AR(1) | 0.51 | 11.72 | 1.00 | -0.17 |  |  |
| DMN-c |  |  |  |  |  |  |  |
| Vis-a / | ARMA(0, 1) | 0.80 | 20.61 |  |  |  |  |
| Attn-dor |  |  |  | 1.00 | 0.19 |  |  |
| Vis-a / Cb | AR(2) | 0.46 | 21.62 | 1.00 | -0.20 | -0.18 |  |
|  |  |  |  |  |  |  |  |
| Vis-b / | AR(2) | 0.25 | 33.74 | 1.00 | -0.19 | -0.15 |  |
| DMN-b |  |  |  |  |  |  |  |
| Vis-b / | ARMA(0, 3) | 0.14 | 43.20 |  |  |  |  |
| DMN-c |  |  |  | 1.00 | 0.22 | 0.26 | 0.34 |
| Vis-b / Cb | AR(2) | 0.53 | 30.25 | 1.00 | -0.04 | -0.26 |  |
|  |  |  |  |  |  |  |  |
| DMN-a / | AR(3) | 0.69 | 44.91 | 1.00 | -0.13 | -0.10 | -0.18 |
| Attn-ven |  |  |  |  |  |  |  |
| DMN-a / | AR(1) | 0.65 | 16.98 | 1.00 | -0.18 |  |  |
| Attn-dor |  |  |  |  |  |  |  |
| DMN-a / | AR(1) | 0.73 | 14.18 | 1.00 | -0.18 |  |  |
| Exec-R |  |  |  |  |  |  |  |
| DMN-a / Sal | ARMA(0, 1) | 0.35 | 20.93 |  |  |  |  |
|  |  |  |  | 1.00 | 0.29 |  |  |
| DMN-b / | AR(1) | 0.14 | 18.71 | 1.00 | -0.25 |  |  |
| Attn-ven |  |  |  |  |  |  |  |
| DMN-b / | ARMA(0, 1) | 0.58 | 19.94 |  |  |  |  |
| Attn-dor |  |  |  | 1.00 | 0.25 |  |  |
| DMN-b / Sal | ARMA(0, 1) | 0.20 | 12.14 |  |  |  |  |
|  |  |  |  | 1.00 | 0.17 |  |  |
| DMN-c / | AR(3) | 0.22 | 43.61 | 1.00 | -0.12 | -0.15 | -0.24 |
| Attn-ven |  |  |  |  |  |  |  |
| DMN-c / | AR(1) | 0.66 | 16.78 | 1.00 | -0.19 |  |  |
| Attn-dor |  |  |  |  |  |  |  |
| DMN-c / Sal | AR(1) | 0.85 | 14.67 | 1.00 | -0.16 |  |  |
|  |  |  |  |  |  |  |  |
| Attn-ven / | AR(1) | 0.92 | 13.91 | 1.00 | -0.15 |  |  |
| Attn-dor |  |  |  |  |  |  |  |
| Attn-ven / | ARMA(0, 1) | 0.32 | 19.42 |  |  |  |  |
| Sal |  |  |  | 1.00 | 0.23 |  |  |
| Attn-dor / | AR(1) | 1.00 | 7.59 | 1.00 | 0.17 |  |  |
| Exec-L |  |  |  |  |  |  |  |
| Attn-dor / | AR(1) | 0.15 | 16.18 | 1.00 | -0.16 |  |  |
| Cb |  |  |  |  |  |  |  |
| Sal / Cb | AR(2) | 0.15 | 29.10 | 1.00 | -0.15 | -0.17 |  |
|  |  |  |  |  |  |  |  |

Properties of the estimated ARMA models for three outcome measures of each RSN are listed. The observed outcome measures are (a) spatial similarity (η^2^), (b) temporal fluctuation magnitude, and (c) BNC. The coefficients of the estimated ARMA model conform to the following equation: $y_{t}+a_{1}y_{t-1}+a_{2}y_{t-2}+a_{3}y_{t-3}=e_{t}+c_{1}e_{t-1}+c_{2}e_{t-2}+c_{3}e_{t-3}$, where autoregressive (AR) coefficients are listed on top rows and moving-average (MA) coefficients are listed on bottom.
