# Supplementary material for: Reproducibility and Temporal Structure in Weekly Resting-State fMRI over a Period of 3.5 Years
Source: PLoS One. 2015 Oct 30;10(10):e0140134. doi: 10.1371/journal.pone.0140134 (PMC4627782; doi:10.1371/journal.pone.0140134)
Supplement: S1 Text — (DOCX) [file pone.0140134.s007.docx]

S1 Text. Detailed explanation of the autoregressive moving average (ARMA) models

S3 Table lists the ARMA models that best describe the autocorrelation structure of the resting state network (RSN) and RSN pair time courses. Additionally, the table reports the corresponding Portmanteau p-values and percent variance explained by the estimated ARMA models. Portmanteau p-values of less than 0.05 indicates that the estimated ARMA model fails to fully describe the autocorrelative properties within a time course – resulting in significant autocorrelation still existing in the time courses even after the estimated ARMA model is regressed out from the original time course. Finally, S3 Table lists the coefficients of the estimated ARMA models, following the structure:

$y_{t}+a_{1}y_{t-1}+a_{2}y_{t-2}+a_{3}y_{t-3}=e_{t}+c_{1}e_{t-1}+c_{2}e_{t-2}+c_{3}e_{t-3}$, ()

where y_t_ is the mean-adjusted series in week *t*, y_t-1_ is the series in the previous week, y_t-2_ is the series from 2 previous weeks, *etc*. Similarly, a_1_ is the lag-1 autoregressive coefficient, a_2_ is the lag-2 autoregressive coefficient, *etc.* e_t_ is defined as the residuals in week t, e_t-1_ is the residuals in the previous week, *etc*. Finally, c_1_ is the first-order moving average (MA) coefficient, c_2_ is the second-order MA coefficient, and so on. For example, a first-order autoregressive and first-order moving average ARMA model, would be indicated as ARMA(1, 1), with an equation of $y_{t}+a_{1}y_{t-1}=e_{t}+c_{1}e_{t-1}$.
